# A maximum-likelihood method to estimate haplotype frequencies and prevalence alongside multiplicity of infection from SNP data

**DOI:** 10.3389/fepid.2022.943625

**Published:** 2022-09-23

**Authors:** Henri Christian Junior Tsoungui Obama, Kristan Alexander Schneider

**Affiliations:** Department of Applied Computer- and Biosciences, University of Applied Sciences Mittweida, Mittweida, Germany

**Keywords:** complexity of infection (COI), multiplicity of infection (MOI), EM-algorithm, drug resistance, malaria, sulfadoxine-pyrimethamine (SP), resistance markers, haplotype phasing

## Abstract

The introduction of genomic methods facilitated standardized molecular disease surveillance. For instance, SNP barcodes in *Plasmodium vivax* and *Plasmodium falciparum* malaria allows the characterization of haplotypes, their frequencies and prevalence to reveal temporal and spatial transmission patterns. A confounding factor is the presence of multiple genetically distinct pathogen variants within the same infection, known as multiplicity of infection (MOI). Disregarding ambiguous information, as usually done in *ad-hoc* approaches, leads to less confident and biased estimates. We introduce a statistical framework to obtain maximum-likelihood estimates (MLE) of haplotype frequencies and prevalence alongside MOI from malaria SNP data, i.e., multiple biallelic marker loci. The number of model parameters increases geometrically with the number of genetic markers considered and no closed-form solution exists for the MLE. Therefore, the MLE needs to be derived numerically. We use the Expectation-Maximization (EM) algorithm to derive the maximum-likelihood estimates, an efficient and easy-to-implement algorithm that yields a numerically stable solution. We also derive expressions for haplotype prevalence based on either all or just the unambiguous genetic information and compare both approaches. The latter corresponds to a biased *ad-hoc* estimate of prevalence. We assess the performance of our estimator by systematic numerical simulations assuming realistic sample sizes and various scenarios of transmission intensity. For reasonable sample sizes, and number of loci, the method has little bias. As an example, we apply the method to a dataset from Cameroon on sulfadoxine-pyrimethamine resistance in *P. falciparum* malaria. The method is not confined to malaria and can be applied to any infectious disease with similar transmission behavior. An easy-to-use implementation of the method as an R-script is provided.

## 1. Introduction

With ever-decreasing costs, genomic/molecular technologies are commonly supporting traditional means of disease surveillance. Rather than clinical data, demographic, or behavioral risk factors, molecular methods provide information on a fine-grained scale that allows to reconstruct sources and routes of disease transmission by reverse engineering or to identify and monitor specific pathogen variants, for instance those associated with drug resistance [cf. (1, 2)]. Improvements in molecular technologies and bioinformatics facilitate the collection of genetic/molecular data on temporal and spatial scales [cf. (3, 4)].

On the epidemiological scale, characterizing pathogen variants on a genomic level, allows, e.g., to identify the emergence of variants resistant to vaccines or therapeutics. Further, by monitoring their prevalence in time and space, paths of transmission can be reconstructed. This might point to weak points in disease control and prevention [cf. (5)]. For the two most relevant species of human malaria, *P. falciparum* and *P. vivax*, even SNP barcodes were developed to standardize molecular surveillance [cf. (6)].

On the individual scale, genomic characterization of pathogen variants can be informative on the clinical pathogenesis of the disease [cf. (7, 8)]. Namely, the presence of drug-resistant variants or the interaction of genetically distinct variants within an infection can influence disease outcomes. Different pathogen variants can assemble in an infection due to co-transmission or independent infective events as a consequence of multiple infectious contacts [cf. (9)]. In malaria this is commonly referred to as multiplicity of infection (MOI) or complexity of infection (COI) (9–12). The concept of MOI and COI is particularly well-recognized in malaria as it is informative on transmission intensities and disease exposure [cf. (11, 13)]. Despite their recognition, MOI or COI are not uniformly defined in the literature [cf. (14)]. Here, we will define MOI in terms of a statistical framework. Although, the concept of MOI and the framework presented here are applicable to a variety of infectious diseases, we have applications to malaria in mind.

A common problem—well recognized in malaria—is the characterization of several different pathogen variants within an infection, because molecular methods do not yield phased genetic information [cf. (15, 16)]. In fact, molecular characterization of pathogen variants (typically haplotypes) and MOI are intrinsically coupled. In practice, two main approaches emerged. The first are *ad-hoc* approaches, which avoid the need to phase molecular information, by disregarding infections with multiple pathogen variants. These approaches are simple to apply at the cost of dismissing the full potential of molecular surveillance. The second are based on formal statistical models. The theoretical background of these methods is sophisticated and applications require some expertise in programming or bioinformatics. For malaria, several such methods have been developed. (Importantly, these methods are in general not restricted to malaria.) The most common ones have a similar underlying statistical framework and are based either on maximum-likelihood (ML) estimation [cf. (14, 17–20)], or Bayesian methods, e.g., classical Bayesian estimation [cf. (21, 22)], or Bayesian hidden Markov models [cf. (23, 24)]. Several methods to estimate MOI and haplotype frequencies are available as software tools [cf. the model comparison in (22)]. For instance, (18) provides a ML method to estimate the distribution of MOI and allele frequencies at one or two genetic markers, assuming that MOI follows either a (conditional) Poisson or (conditional) negative binomial distribution. In the case of the conditional Poisson distribution the method was further developed by (20, 25), who also provided efficient implementations. A user-friendly implementation which allows flexible data handling is provided by the R package MLMOI [cf. (26)]. A bias-corrected ML approach was provided by (14). MalHaploFreq [cf. (27)] uses a ML approach to estimate the distribution of haplotypes characterized by up to three biallelic loci (e.g., SNPs) and the distribution of MOI, which is assumed to follow either a Poisson, conditional Poisson, or negative binomial distribution. This approach was generalized to an arbitrary number of SNPs for the cases of the Poisson and conditional Poisson distribution [cf. (28)]. It makes use of the expectation-maximization algorithm (EM algorithm) to derive the ML estimates. However, the algorithm was neither derived in an explicit and efficient way, nor was an implementation made available. A different ML approach to estimate haplotype frequencies based on several approximations that guarantee numerical feasibility was suggested [cf. (29)]. This method also provides estimates for MOI, but based on several simplifications. Bayesian approaches include the method based on Gibbs sampling by (22) to estimate haplotype frequencies from SNP data including an error model, the Metropolis-Hastings algorithm of (30, 31) to estimate frequencies of haplotypes which are not restricted to biallelic loci. However, this approach requires heuristic estimates of MOI. For biallelic loci, the program COIL offers a classical Bayesian approach to estimate MOI (COI) [cf. (32)]. THE REAL McCOIL [cf. (33)] is a generalization based on the Metropolis-Hastings algorithm. It estimates MOI and minor-allele frequencies at uncorrelated SNPs in two different ways. Importantly, ML-based methods and Bayesian methods should yield consistent results, as both involve the likelihood function. In the strict sense, ML approaches provide point estimates for parameters, whereas Bayesian methods provide posterior distributions for parameters of interest. Agreement should be particularly strong if the prior distribution is uninformative, or if the prior distribution gives substantial weight to the true parameters and the data set is representative. Discrepancies between the methods are expected if: (i) the data is an “outlier” and the prior gives substantial weight to the true parameters (in which case, essentially all information is excerpted from the prior, which is more reliable than the posterior); (ii) the data is reliable, but the prior gives too much weight to the wrong parameters.

As genomics data is becoming more common in diseases like malaria, methods capable to handle such data became popular. For example, an approach to estimate MOI from deep-sequencing data is provided in (34). Moreover, methods considering relatedness of pathogen variants within infections become increasingly popular [cf. (35–40)]. Models such as DeploidIBD [cf. (40)] estimate the number and proportions of haplotypes in an infection alongside their identity-by-descent (IBD) profiles.

Here, we use a ML approach to estimate the frequencies of haplotypes, determined by *n* biallelic loci, and the distribution of MOI, assuming a conditional Poisson distribution. As with related methods, MOI is defined as the number of super-infections (i.e., independent infectious events during the course of the disease under the assumption of no co-transmission of pathogen variants). The proposed method is intended for a moderate number of loci, i.e., *n* should not be so large that individual haplotypes will be characterized. In particular, we employ the EM algorithm as in (28). While the general form provided by (28) is easily derived, a more explicit form is combinatorically involved and complicated. Here, we provide such an explicit form. This is the foundation of an efficient implementation of the algorithm. Such an implementation is provided as an easy-to-use R script. Importantly, we derive expressions for prevalence of haplotypes, i.e., the probability that a given haplotype occurs in an infection. Prevalence is mediated by MOI, i.e., it is derived from the haplotype frequency distribution and the distribution of MOI. If primarily interested in disease outcomes rather than the population genetics of the pathogen, prevalence is more relevant than the frequency distribution of haplotypes. Prevalence is notoriously difficult to estimate, especially from unphased molecular data. Namely, a statistical model, as the one presented here, is required for its estimation. Without such a model, *ad-hoc* estimates can be made from samples without ambiguity regarding haplotype phasing [e.g., as done in some of the analyses in (41)]. Such estimates are however biased. We assess the performance of the estimator of MOI, frequencies, prevalence, and *ad-hoc* approximations of prevalence in terms of bias and variance by numerical simulations.

As an example, we apply the method to estimate the frequency of malaria haplotypes associated with resistance against sulfadoxine-pyrimethamine (SP). Specifically, we apply the method to molecular data obtained from malaria-positive blood samples collected in Cameroon at two time points [cf. (42)].

We start with a formulation of the underlying statistical framework and a clear definition of MOI. Readers not focused on mathematical rigor shall feel free to move directly to the result section. Formal proofs and derivations are provided in the Mathematical Appendix.

## 2. Methods

A formal description of the statistical model is presented here. The model extends the method of (18), further developed by (25) to estimate multiplicity of infection (MOI) defined as the number of super-infections (i.e., independent infectious events during the course of the disease under the assumption of no co-transmissions/co-infections) and allele frequencies at a single-marker locus. Here, we extend the method to an arbitrary number of marker loci each with two alleles, e.g., single nucleotide polymorphisms (SNPs) as used in *P. vivax* or *P. falciparum* barcodes [cf. (6)], to estimate the haplotypes frequency distribution and MOI. As pointed out in (43), the assumption of no co-infections is not too strict. More precisely, the model approximately also holds if co- and super-infections occur.

### 2.1. Statistical model

Consider pathogen haplotypes, denoted ***h***, characterized by *n* biallelic markers. At each locus, the wildtype allele is coded by 0 and the mutant allele by 1. Hence, a haplotype is represented by a 0-1-vector indicating its allelic configuration, i.e., ***h*** = (*h*_1_, …, *h*_*n*_), with *h*_*k*_ ∈ {0, 1}. A total of *H* = 2^*n*^ haplotypes are possible. The set of all possible haplotypes is thus given by ***h*** ∈ H = {0, 1}^*n*^.

Each haplotype ***h*** (0-1-vector) corresponds to a binary representation of the numbers 1, …, 2^*n*^, namely to ${\left[h\right]}_{2}:=1+\overset{n}{\underset{l=1}{\sum }}h_{l}2^{l-1}$. (As an example for *n* = 4 the haplotype (1, 0, 0, 1) corresponds to the number 1+1·2^0^+0·2^1^+0·2^2^+1·2^3^ = 1+1+8 = 10). We order haplotypes according to that representation.

The frequency of haplotype ***h***, denoted by *p*_***h***_, is its relative abundance in the pathogen population (assessed at a particular census point). For example, in the case of malaria, the frequency of a haplotype is its relative abundance in the sporozoite population in the mosquitoes' salivary glands (20). Collectively, the frequencies form the vector ***p***: = (*p*_***h***_)_***h***∈H_ = (*p*_1_, …, *p*_*H*_), where *p*_*k*_ = *p*_***h***_ if [***h***]_2_ = *k*. In practice, *p*_*k*_ = 0 for several haplotypes, since not all 2^*n*^ possible haplotypes will be present in the pathogen population.

It is assumed that at each infective event, exactly one pathogen haplotype is transmitted. However, individuals can get (super-) infected several times during one disease episode. The number of (super-) infections during one disease episode is referred here to as multiplicity of infection (MOI). We treat the terms complexity of infection (COI) and MOI synonymously here. The term super-infections refers here to independent infective events without co-transmissions, i.e., only one pathogen variant is transmitted. In contrast, a co-infection is one infective event during which several pathogen variants are co-transmitted. However, the model is still approximately applicable if co-transmissions occur [cf. (43)].

Assuming that infections are rare and independent, MOI follows a Poisson distribution. When considering only disease-positive individuals, MOI follows a conditional (or positive) Poisson distribution. Thus, the probability to be (super-) infected exactly *m* times (MOI = *m*) is given by [see (25)]


(1a) $$\begin{array}{l} \kappa_{m}=\frac{1}{e^{\lambda }-1}\frac{\lambda^{m}}{m!},\;m=1,2,3,\ldots . \end{array}$$

Note that, a zero-inflated Poisson distribution [cf. (44)] yields the same conditional Poisson distribution. Therefore, *m* ~ CPoiss(λ), where λ is the parameter characterizing the conditional Poisson distribution.

The probability generating function (PGF) of the conditional Poisson distribution is given by


(1b) $$\begin{array}{l} G\left(z\right):=𝔼\left[z^{m}\right]=\overset{\infty }{\underset{m=1}{\sum }}\kappa_{m}z^{m}=\frac{e^{\lambda z}-1}{e^{\lambda }-1}, \end{array}$$

and the mean MOI is given by (see 25)


(1c) $$\begin{array}{l} \psi :=𝔼\left[m\right]=\frac{\lambda }{1-e^{-\lambda }}. \end{array}$$

At each infective event, exactly one haplotype, randomly chosen from the pathogen population, is transmitted to the host. Given, an individual is super-infected *m* times (MOI = *m*), the process of infection corresponds to multinomially sampling from the pathogen population. If *m*_***h***_ is the number of times an individual was infected with haplotype ***h*** (necessarily $|m|:=\underset{h\in H}{\sum }m_{h}=m_{1}+\ldots +m_{H}=m$), the infection is subsumed by the vector ***m***: = (_*m*_***h***_)***h*** ∈ H_ = (*m*_1_, …, *m*_*H*_). Therefore, given MOI *m*, infection ***m*** (with |***m***| = *m*) occurs with probability


(2) $$\begin{array}{l} P\left(m|m\right)=\left(\begin{array}{l} m\\ m \end{array}\right)p^{m}, \end{array}$$

where $\left(\begin{array}{l} m\\ m \end{array}\right)=\frac{m!}{m_{1}!\ldots m_{H}!}$, and $p^{m}=p_{1}^{m_{1}}\ldots p_{H}^{m_{H}}$.

**Figure 1:**
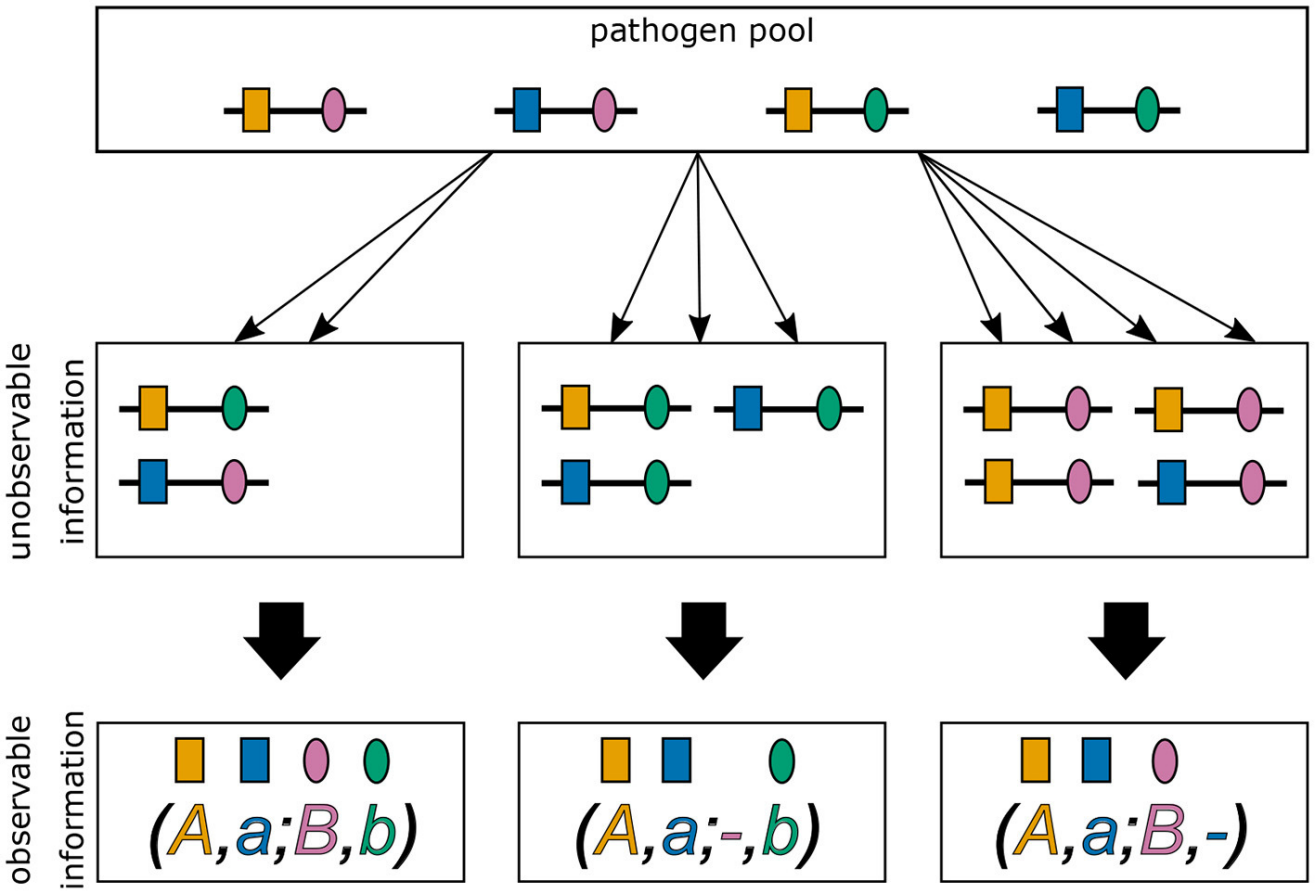
Ambiguity in haplotype information for two biallelic loci. Illustrated are three different infections from the same pathogen population. The first infection **(middle left)** describes a super-infection with two haplotypes, i.e., MOI = 2. The corresponding observation **(bottom left)** provides only unphased (i.e., ambiguous) haplotype information. It is impossible to reconstruct with certainty the haplotypes actually present in the infection and the corresponding MOI. The second infection **(middle)**, illustrates a super-infection with two haplotypes transmitted one and two times, respectively, i.e., MOI = 3. From the observed information, the haplotypes present in the infections can be unambiguously phased. However, MOI remains unknown. The last infection **(middle right)** is similar to the second, however with MOI = 4.

In practice, the vector ***m*** and even MOI *m* are unobservable from a clinical specimen. In addition, assays to determine genetic information usually do not yield full haplotype information, i.e., if multiple different haplotypes are present within an infection, assays yield ambiguous genetic information due to the lack of phasing (cf. Figure 1) (15). It is assumed here, that only the absence/presence of alleles at every locus is assessable. [Notably, in the case of phased data, haplotypes as defined here, would be equivalent to alleles at a single multi-allelic marker locus and can be analyzed with the methods of (18, 20, 25).]

We denote allelic information of an infection by a vector ***x*** = (*x*_1_, …, *x*_*n*_), where *x*_*k*_ is the set of alleles detected at locus *k* in the sample. It is assumed that all alleles that are present in an infection are actually detected and that alleles are not erroneously detected. Therefore, *x*_*k*_ equals one of the sets {0}, {1}, or {0, 1}, corresponding to, respectively, the presence of the wildtype, the mutant, or both alleles at locus *k*. The set of all possible observations is O: = ({{0}, {1}, {0, 1}})^*n*^. There are a total of 3^*n*^ possible observations. Several different infections ***m*** can yield the same observation ***x*** (***m*** → ***x***). The set of all infections with MOI equal to *m* which yield observation ***x*** is denoted by


(3a) $$\begin{array}{l} M_{x}^{\left(m\right)}:=\left\{m|m\to x,|m|=m\right\}. \end{array}$$

Furthermore, we denote the set of all haplotypes which are compatible with observation ***x*** (i.e., the set of all haplotypes that could potentially be present in the underlying infection) by


(3b) $$\begin{array}{l} A_{x}:=\left\{h=\left(h_{1},\ldots ,h_{n}\right)|h_{k}\in x_{k}\text{ for all }k\right\}. \end{array}$$

Let us denote the set of sub-observations of observation ***x***, i.e., all observations with at most the same alleles detected at each locus as in ***x*** (cf. Supplementary Figure 1), by


(3c) $$\begin{array}{l} A_{x}:=\left\{y=\left(y_{1},\ldots ,y_{n}\right)|y_{k}\subseteq x_{k}\text{ for all }k\right\}. \end{array}$$

If ***y*** is a sub-observation of ***x***, i.e., ***y*** ∈ A_***x***_, we write ***y***≼***x***. Note that “≼” defines a partial order on the set of possible observations. We further define the proper sub-observation ***y*** of ***x*** by


(4) $$\begin{array}{l} y≺x:\Leftrightarrow y≼x\wedge x\neq y. \end{array}$$

Using this notation the set $M_{x}^{\left(m\right)}$ is rewritten as


(5) $$\begin{array}{l} M_{x}^{\left(m\right)}=\{m|m_{h}=0\text{ if }h\notin A_{x},\\ \;\left|m\right|=m\}\backslash \underset{y≺x}{\cup }\left\{m|m_{h}=0\text{ if }h\notin A_{y}\left|m\right|=m\right\}, \end{array}$$

where *A*_***y***_ is defined as (3) but for the proper sub-observation ***y*** rather than for the original observation ***x***. Given an infection with MOI *m*, the probability of observing ***x*** is


(6a) $$\begin{array}{l} P\left(x|m\right)=\frac{P\left(x,m\right)}{\kappa_{m}}. \end{array}$$

Therefore,


(6b) $$\begin{array}{l} \begin{array}{c} P\left(x,m\right)=P\left(M_{x}^{\left(m\right)}\right)=\underset{m\in M_{x}^{\left(m\right)}}{\sum }P\left(m\right)=\underset{m\in M_{x}^{\left(m\right)}}{\sum }P\left(m|m\right)\kappa_{m}\\ \;=\kappa_{m}\underset{m\in M_{x}^{\left(m\right)}}{\sum }\left(\begin{array}{l} m\\ m \end{array}\right)p^{m}. \end{array} \end{array}$$

The probability of observation ***x*** becomes


(6c) $$\begin{array}{l} P\left(x\right)=\overset{\infty }{\underset{m=1}{\sum }}P\left(x,m\right)=\overset{\infty }{\underset{m=1}{\sum }}\kappa_{m}\underset{m\in M_{x}^{\left(m\right)}}{\sum }\left(\begin{array}{l} m\\ m \end{array}\right)p^{m}. \end{array}$$

Henceforth, to simplify the notation we will use *P*_***x***_ to denote the probability of observing ***x*** instead of *P*(***x***). By using (3a) and the inclusion-exclusion principle the inner sum on the right-hand side of (6c) can be rewritten as


(7a) $$\begin{array}{l} \underset{m\in M_{x}^{\left(m\right)}}{\sum }\left(\begin{array}{l} m\\ m \end{array}\right)p^{m}=\underset{\begin{array}{c} m:|m|=m\\ m_{h}=0\text{ if }h\notin A_{x} \end{array}}{\sum }\left(\begin{array}{l} m\\ m \end{array}\right)p^{m}\\ \;+\underset{y≺x}{\sum }{\left(-1\right)}^{\overset{n}{\underset{k=1}{\sum }}\left(|x_{k}|-|y_{k}|\right)}\underset{\begin{array}{c} m:|m|=m\\ m_{h}=0\text{ if }h\notin A_{y} \end{array}}{\sum }\left(\begin{array}{l} m\\ m \end{array}\right)p^{m}, \end{array}$$

where |*x*_*k*_| and |*y*_*k*_| are, respectively, the cardinals of *x*_*k*_, and *y*_*k*_. Let *N*_***x***_ denote the number of loci in observation ***x*** at which both alleles were detected, i.e., *N*_***x***_ = |{*k*| |*x*_*k*_| = 2}|. The number of loci with a single allele detected is then *n*−*N*_***x***_. Hence, the number of alleles detected in observation ***x*** is given by 2*N*_***x***_ + *n* − *N*_***x***_ = *n* + *N*_***x***_. We hence obtain


(7b) $$\begin{array}{l} \overset{n}{\underset{k=1}{\sum }}\left(|x_{k}|-|y_{k}|\right)=N_{x}-N_{y}, \end{array}$$

and


(7c) $$\begin{array}{l} \begin{array}{c} \underset{m\in M_{x}^{\left(m\right)}}{\sum }\left(\begin{array}{l} m\\ m \end{array}\right)p^{m}=\underset{\begin{array}{c} m:|m|=m\\ m_{h}=0\text{ if }h\notin A_{x} \end{array}}{\sum }\left(\begin{array}{l} m\\ m \end{array}\right)p^{m}\\ \;+\underset{y≺x}{\sum }{\left(-1\right)}^{N_{x}-N_{y}}\underset{\begin{array}{c} m:|m|=m\\ m_{h}=0\text{ if }h\notin A_{y} \end{array}}{\sum }\left(\begin{array}{l} m\\ m \end{array}\right)p^{m}\\ \;=\underset{y≼x}{\sum }{\left(-1\right)}^{N_{x}-N_{y}}\underset{\begin{array}{c} m:|m|=m\\ m_{h}=0\text{ if }h\notin A_{y} \end{array}}{\sum }\left(\begin{array}{l} m\\ m \end{array}\right)p^{m}\\ \;=\underset{y\in A_{x}}{\sum }{\left(-1\right)}^{N_{x}-N_{y}}\underset{\begin{array}{c} m:|m|=m\\ m_{h}=0\text{ if }h\notin A_{y} \end{array}}{\sum }\left(\begin{array}{l} m\\ m \end{array}\right)p^{m}. \end{array} \end{array}$$

Therefore, the probability of observing ***x*** in (6c) becomes


(8a) $$\begin{array}{l} P_{x}=\overset{\infty }{\underset{m=1}{\sum }}\kappa_{m}\underset{y\in A_{x}}{\sum }{\left(-1\right)}^{N_{x}-N_{y}}\underset{\begin{array}{c} m:|m|=m\\ m_{h}=0\text{ if }h\notin A_{y} \end{array}}{\sum }\left(\begin{array}{l} m\\ m \end{array}\right)p^{m}. \end{array}$$

By the multinomial theorem, we have


(8b) $$\begin{array}{l} \underset{\begin{array}{c} m:|m|=m\\ m_{h}=0\text{ if }h\notin A_{y} \end{array}}{\sum }\left(\begin{array}{l} m\\ m \end{array}\right)p^{m}={\left(\underset{h\in A_{y}}{\sum }p_{h}\right)}^{m}. \end{array}$$

Using the PGF (8a) becomes


(8c) $$\begin{array}{l} \begin{array}{c} P_{x}=\overset{\infty }{\underset{m=1}{\sum }}\kappa_{m}\underset{y\in A_{x}}{\sum }{\left(-1\right)}^{N_{x}-N_{y}}{\left(\underset{h\in A_{y}}{\sum }p_{h}\right)}^{m}\\ \;=\underset{y\in A_{x}}{\sum }{\left(-1\right)}^{N_{x}-N_{y}}\overset{\infty }{\underset{m=1}{\sum }}\kappa_{m}{\left(\underset{h\in A_{y}}{\sum }p_{h}\right)}^{m}\\ \;=\underset{y\in A_{x}}{\sum }{\left(-1\right)}^{N_{x}-N_{y}}G\left(\underset{h\in A_{y}}{\sum }p_{h}\right). \end{array} \end{array}$$

This probability depends on the model parameters λ, which appears in the PGF, and the vector of haplotype frequencies ***p***. Hence, the parameter space of the model is


(9) $$\begin{array}{l} \Theta :={\mathbb{R}}^{+}\times S_{H}=\left\{\left(\lambda ,p\right)|\lambda >0\text{ and }p\in S_{H}\right\}, \end{array}$$

where, $S_{H}:=\left\{\left(p_{1},\ldots ,p_{H}\right)|\overset{H}{\underset{k=1}{\sum }}p_{k}=1\text{ and }p_{k}\geq 0,\text{ for all }k\right\}$ is the *H*−1-dimensional simplex.

The true parameter vector ***θ*** = (λ, ***p***) is unknown and has to be estimated from empirical data. Assume a dataset X consisting of *N* observations ***x***^(1)^, …, ***x***^(*N*)^, where the notation $x^{\left(j\right)}=\left(x_{1}^{\left(j\right)},\ldots ,x_{n}^{\left(j\right)}\right)$ is used for the *j*th observation. For the dataset X, let *n*_***x***_ be the number of times observation ***x*** is made. Naturally,


$$\begin{array}{l} \underset{x\in O}{\sum }n_{x}=N. \end{array}$$

Using (8c), the likelihood function of the parameter ***θ*** = (λ, ***p***) given the data X is given by


(10) $$\begin{array}{l} L_{X}\left(\theta \right)=\overset{N}{\underset{j=1}{\prod }}P_{x^{\left(j\right)}}=\underset{x\in O}{\prod }(\underset{y\in A_{x}}{\sum }{\left(-1\right)}^{N_{x}-N_{y}}G(\underset{h\in A_{y}}{\sum }p_{h}){)}^{n_{x}}. \end{array}$$

Hence, the log-likelihood function becomes


(11) $$\begin{array}{l} \ell_{X}\left(\theta \right)=\log\left(L_{X}\left(\theta \right)\right)\\ \;=\underset{x\in O}{\sum }n_{x}\log\left(\underset{y\in A_{x}}{\sum }{\left(-1\right)}^{N_{x}-N_{y}}G(\underset{h\in A_{y}}{\sum }p_{h})\right). \end{array}$$

To obtain the maximum-likelihood estimate (MLE) $\hat{\theta }=\left(\hat{\lambda },\hat{p}\right)$ the log-likelihood function needs to be maximized. The complexity of the log-likelihood function does not permit a closed solution, and must be maximized numerically. For this purpose the expectation-maximization (EM)-algorithm will be used (17). This will be discussed in Section 3.2.

#### 2.1.1. Confidence intervals

To ascertain uncertainty of the estimates, confidence intervals (CIs) can be derived. A straightforward approach is to derive bootstrap CIs. The simplest type of boostrap CIs are the non-parametric percentile CIs [cf. (45), Chapter 13]. To obtain a (1 − α)% bootstrap CIs from a dataset X of sample size *N*, we sample *B* (e.g., *B* = 10, 000) datasets X_1_, …, X_*B*_, each of sample size *N* with replacement from X. For each dataset X_*b*_, we obtain the MLEs ${\hat{\theta }}^{\left(b\right)}$. For the desired parameter θ_*k*_, the $\frac{\alpha }{2}\%$ and $\left(1-\frac{\alpha }{2}\right)\%$ percentiles, ${\hat{\theta }}_{k,\frac{\alpha }{2}}^{*}$ and ${\hat{\theta }}_{k,\left(1-\frac{\alpha }{2}\right)}^{*}$, respectively, are determined from the sequence ${\hat{\theta }}_{k}^{\left(1\right)},\ldots ,{\hat{\theta }}_{k}^{\left(B\right)}$. The (1−α)% CI is then given by


(12) $$\begin{array}{l} \left({\hat{\theta }}_{k,\frac{\alpha }{2}}^{*},{\hat{\theta }}_{k,1-\frac{\alpha }{2}}^{*}\right). \end{array}$$

Clearly, more advanced bootstrap CIs, e.g., bias-corrected and accelerated (BCa) boostsrap CIs [cf. (45), Chapter 14] or parametric bootstrap CIs [cf. (45), Chapter 12] can be calculated similarly.

#### 2.1.2. Assessing bias and variance of the estimator

MLEs have desirable asymptotic properties, i.e., for large sample size. In practice, sample size is often limited, and the quality of the estimator needs to be investigated under finite sample sizes. Because no explicit solution exists for the MLE, its performance in terms of bias and variance needs to be investigated by numerical simulations.

Bias and variance of the MLE will be affected by: (i) sample size *N*; (ii) the number of considered loci *n*, i.e., the genetic architecture; (iii) the value of the MOI parameter λ; (iv) the frequency distribution of haplotypes ***p***.

To investigate the properties of the MLE for a representative range of parameters we proceeded as follows (parameters used in the simulation study are described below and summarized in Table 1). For a set of parameters (*N*, *n*, λ, ***p***) we generated *K* = 100, 000 datasets X_1_, …, X_*K*_ of size *N* according to the model (8c). For each dataset X_*k*_, the MLE $\left({\hat{\theta }}_{k}\right)=\left({\hat{\lambda }}_{k},{\hat{p}}^{\left(k\right)}\right)$ was calculated. From each ${\hat{\lambda }}_{k}$ the mean MOI ${\hat{\psi }}_{k}$ was calculated according to (1c). The bias and variance of the mean MOI ψ were estimated as


(13a) $$\begin{array}{l} \mathrm{bias}\left(\hat{\psi }\right)=\bar{\psi }-\psi , \end{array}$$

and


(13b) $$\begin{array}{l} \mathrm{Var}\left(\hat{\psi }\right)=\frac{1}{K-1}\overset{K}{\underset{k=1}{\sum }}{\left({\hat{\psi }}_{k}-\bar{\psi }\right)}^{2}, \end{array}$$

where


(13c) $$\begin{array}{l} \bar{\psi }=\frac{1}{K}\overset{K}{\underset{k=1}{\sum }}{\hat{\psi }}_{k}. \end{array}$$

**Table 1:** Summary of model parameters chosen for the simulations to assess the estimator's performance.

**Parameter**	**Description**	**Value**		
*K*	Number of simulated datasets	100, 000		
*n*	Number of loci markers	2, 5, 10		
*N*	Sample size	50, 100, 150, 200, 500		
λ	MOI parameter	0.1, 0.25, 0.5, 1, 1.5, 2, 2.5		
			**Symmetric**	**Skewed**
***p***	Hapl. freq. (simulated data)	*n* = 2:	$p_{1}=\ldots =p_{4}=\frac{1}{4}$	*p*_1_ = 0.7,
				*p*_2_ = … = *p*_4_ = 0.1
		*n* = 5:	$p_{1}=\ldots =p_{32}=\frac{1}{32}$	*p*_1_ = 0.7,
				$p_{2}=\ldots =p_{32}=\frac{0.3}{31}$

Freq., Frequency; Hapl., Haplotype.

To allow comparisons between different parameter ranges it is more appropriate to consider the relative bias and coefficient of variation which are independent of the scale, i.e.,


(14a) $$\begin{array}{l} \frac{\mathrm{bias}\left(\hat{\psi }\right)}{\psi }, \end{array}$$

and


(14b) $$\begin{array}{l} \frac{\sqrt{\mathrm{Var}\left(\hat{\psi }\right)}}{\psi }. \end{array}$$

For each haplotype frequency *p*_***h***_, bias and variance were defined in the same way with obvious modifications.

##### 2.1.2.1. Genetic architecture

Considering the number of biallelic loci, for the simulations we assumed *n* = 2, 5 to perform systematic investigations of the estimator. The number of possible haplotypes was then 4 and 32, respectively, for *n* = 2, 5. As the number of loci increases due to the curse of dimensionality it becomes too exhaustive to perform systematic investigations. Hence, in addition, we chose two specific distributions for *n* = 10, which correspond to distributions of drug-resistant haplotypes which were previously empirically estimated. Importantly, the method is not limited to just 10 loci.

##### 2.1.2.2. MOI parameter

Concerning the MOI parameter we chose λ = 0.1, 0.25, 0.5, 1, 1.5, 2, 2.5, corresponding to a mean MOI ψ = 1.05, 1.13, 1.27, 1.58, 1.93, 2.31, 2.72. In the case of malaria, this corresponds to low transmission ψ < 1.27, intermediate transmission 1.27 ≤ ψ < 1.93 and high transmission ψ ≥ 1.93 (14).

##### 2.1.2.3. Haplotype frequency distribution

The following haplotype frequency distributions ***p*** were chosen. First, a completely uniform (balanced) distribution was chosen, i.e., each of the *H* = 2^*n*^ haplotype had the same frequency,


(15a) $$\begin{array}{l} p_{1}=\ldots =p_{H}=\frac{1}{H}. \end{array}$$

Second, a skewed distribution with one predominant haplotype was chosen. The frequency of the predominant haplotype was chosen to be 70%, while the remaining haplotypes all had the same frequency. In particular, we chose


(15b) $$\begin{array}{l} p_{1}=0.7,p_{2}=\ldots =p_{H}=\frac{0.3}{H-1}. \end{array}$$

For *n* = 2 this yielded, *p*_1_ = 0.7, *p*_2_ = *p*_3_ = *p*_4_ = 0.1 and for *n* = 5 loci *p*_1_ = 0.7, *p*_2_ = … = *p*_32_ = 0.0097.

Third, we chose specific empirical distributions for the case *n* = 10. The reason is that the dimension of the parameter space becomes high (*H* = 1, 024), but most haplotypes will not be realized in a population. Specifically, we assumed two haplotype frequency distributions that correspond to empirically estimated distributions of *P. falciparum* malaria haplotypes. These haplotypes were characterized by *n* = 10 SNPs associated with resistance to sulfadoxine-pyrimethamine (SP). The two haplotype frequency distributions were estimated from a population in Siaya County, Kenya, respectively, in 2005 and 2010 (see (46)).

In 2005, the frequencies of detected haplotypes were *p*_2_ = 0.055, *p*_5_ = 0.016, *p*_6_ = 0.171, *p*_11_ = 0.015, *p*_13_ = 0.024, *p*_14_ = 0.719, respectively, while in 2010 they were *p*_1_ = 0.007, *p*_3_ = 0.015, *p*_6_ = 0.084, *p*_7_ = 0.006, *p*_14_ = 0.791, *p*_15_ = 0.007, *p*_16_ = 0.009, *p*_19_ = 0.081, respectively (see Table 2).

**Table 2:** Frequencies of SP-resistant haplotypes from the Kenyan data used for simulation study.

**Haplotype**		**Frequency ***p*****	
		**Years**	
***dhfr***	***dhps***	**2005**	**2010**
NC**N**	S**GE**A	−	0.007
N**RN**	S**GE**A	0.055	−
**I**C**N**	SAKA	−	0.015
**I**C**N**	S**G**KA	0.016	−
**I**C**N**	S**GE**A	0.171	0.084
**I**C**N**	**A**AKA	−	0.006
**IRN**	SAKA	0.015	−
**IRN**	S**G**KA	0.024	−
**IRN**	S**GE**A	0.719	0.791
**IRN**	S**GEG**	−	0.007
**IRN**	**A**AKA	−	0.009
**IRN**	**AGE**A	−	0.081

The first column shows the amino acid sequence at codons 51, 59, and 108 at the *dhfr* locus and the second column shows the amino acid sequence at codons 436, 437, 540, and 581 at the *dhps* locus.

##### 2.1.2.4. Sample size

Sample size is crucial to the performance of an estimator. To evaluate the effect of sample size, we constructed datasets of size *N* = 50, 100, 150, 200, 500. In malaria *N* = 50 − 150 are typical sample sizes. The large sample size *N* = 500, which is becoming more common in malaria, but might still be infeasible for low transmission areas. Nevertheless, considering *N* = 500 helps understand the asymptotic behavior of the estimator.

We used R (47) to implement the simulation study and create the graphical outputs. The code is available at: https://github.com/Maths-against-Malaria/MultiLociBiallelicModel.git.

### 2.2. Data application

As an application, we estimated the frequency of malaria haplotypes associated with resistance against SP. The data was taken from (42, 48, 49) and is described there in detail. In short, it was collected in Yaoundé, Cameroon in 2001/2002 and 2004/2005. Mutations at codons 51, 59, 108, and 164 at the *dhfr* locus on chromosome 4 and 436, 437, 540, 581, and 613 at the *dhps* locus on chromosome 8 were determined either by direct sequencing or pyrosequencing. Due to missing data, we included 165 samples from 2001/2002 and 165 samples from 2004/2005.

## 3. Results

For molecular surveillance of parasite haplotypes, obtaining adequate estimates of haplotype frequencies is crucial. From a clinical point of view, the occurrence of particular haplotypes in infections, i.e., the prevalence of haplotypes, is more relevant. Importantly, frequency and prevalence are not the same, as the latter is mediated by MOI [cf. (43)]. First, we clarify the relationship between frequency, prevalence, and MOI. Second, an efficient algorithm for estimating MOI, haplotype frequencies, and prevalence is provided. Finally, the properties of the estimator are investigated numerically.

### 3.1. Prevalence and similar quantities

The frequency of haplotype ***h***, i.e., *p*_***h***_, defines its relative abundance in the pathogen population. According to the underlying model, *p*_***h***_ is the probability that haplotype ***h*** is transmitted at a given infective event. However, several infective events (super-infections) can cause an infection, so that the probability that haplotype ***h*** is transmitted at any infective event exceeds *p*_***h***_. The probability that haplotype ***h*** is present in an infection is called its prevalence.

Typically, molecular assays do not provide phased information. Hence, if several haplotypes are present within an infection, it is ambiguous which haplotypes are actually infecting (cf. Figure 1).

Observations that carry only one haplotype are called single infections, i.e., an observation ***x*** is a single infection if |*x*_*k*_| = 1 ∀*k*. Molecular information from single infections are unambiguous. However, even for single infections MOI is unobservable (since it is unclear how many times the host was super-infected with the same haplotype).

Infections with two or more distinct haplotypes are called multiple infections. The resulting molecular information is ambiguous, except in the case of exactly two super-infecting haplotypes that differ at only one locus. We call such super-infections unambiguous multiple infections. Namely, $\tilde{x}$ is an unambiguous multiple infection if there exist a unique locus *k* such that $|{\tilde{x}}_{k}|=2$ and $|{\tilde{x}}_{l}|=1\;\forall l\neq k$. Clearly, MOI—as for every sample—is unobservable.

Since haplotype information is ambiguous in multiple infections, to assess the prevalence of haplotypes sometimes only unambiguous samples are considered in practice. Here, we first define prevalence in general (unobservable prevalence). Second, we derive the probability to observe a given haplotype in unambiguous samples (conditional prevalence).

#### 3.1.1. Prevalence

Since haplotype information is typically unavailable from molecular assays, haplotypes are per se not observable in molecular samples. To emphasize this fact we call the probability that a haplotype occurs in an infection “unobservable prevalence.” The unobservable prevalence of ***h*** is denoted by *q*_***h***_ and the probability that haplotype ***h*** does not occur in infection by *q*_−***h***_ = 1 − *q*_***h***_. The unobservable prevalence is hence


(16a) $$\begin{array}{l} q_{h}=1-q_{-h}=1-\overset{\infty }{\underset{m=1}{\sum }}\kappa_{m}\underset{\begin{array}{c} m:|m|=m\\ m_{h}=0 \end{array}}{\sum }\left(\begin{array}{l} m\\ m \end{array}\right)p^{m}. \end{array}$$

By using the multinomial theorem one obtains


(16b) $$\begin{array}{l} \underset{\begin{array}{c} m:|m|=m\\ m_{h}=0 \end{array}}{\sum }\left(\begin{array}{l} m\\ m \end{array}\right)p^{m}=(\underset{i\in H}{\sum }p_{i}-p_{h}{)}^{m}={\left(1-p_{h}\right)}^{m}. \end{array}$$

This yields


(16c) $$\begin{array}{l} q_{h}=1-\overset{\infty }{\underset{m=1}{\sum }}\kappa_{m}{\left(1-p_{h}\right)}^{m}=1-G\left(1-p_{h}\right)\\ \;=1-\frac{e^{\lambda \left(1-p_{h}\right)}-1}{e^{\lambda }-1}. \end{array}$$

Hence, prevalence is derived readily from the MOI parameter λ and the frequencies. Given the frequency of haplotype ***h*** its unobservable prevalence increases with increasing MOI (increasing λ).

The unobservable prevalence *q*_***h***_ always exceeds the frequency *p*_***h***_ of haplotype ***h***. The higher transmission intensities, the more does prevalence exceed frequency. This is illustrated in **Figures 10**–**13**. In the limit of λ → 0, i.e., every infection is a single infection with MOI *m* = 1, prevalence and frequency coincide. Distinguishing between frequency and prevalence is hence particularly important in areas of seasonal disease transmission, where the parameter λ will fluctuate between seasons.

#### 3.1.2. Conditional prevalence

Because of ambiguity of haplotype information in multiple infections, it is impossible to identify the number of samples containing haplotype ***h*** in a dataset. In practice, often only unambiguous samples are considered, to determine prevalence. Here, we derive the corresponding quantity in the underlying framework, i.e., the prevalence of haplotype ***h***, conditioned on observing only unambiguous data. The quantity is referred to as “conditional prevalence.”

We denote the set of all possible unambiguous observations by $\tilde{O}$. The conditional prevalence is


(17) $$\begin{array}{l} r_{h|\tilde{O}}:=P\left(h|\tilde{O}\right)=\frac{P\left(h,\tilde{O}\right)}{P\left(\tilde{O}\right)}=\frac{r_{h}}{P\left(\tilde{O}\right)}, \end{array}$$

where $r_{h}:=P\left(h,\tilde{O}\right)$ is the probability to observe haplotype ***h*** in an unambiguous observation, and $P\left(\tilde{O}\right)$ is the probability of unambiguous observations. For each haplotype ***h***, let *U*_***h***_ be the set of all haplotypes ***i***, which yield unambiguous observations with ***h*** (i.e., if only ***h*** and ***i*** are present in an infection, it is an unambiguous infection). Note that there are exactly *n* haplotypes ***i*** such that ***i*** ∈ *U*_***h***_. Formally, we have


(18) $$\begin{array}{l} U_{h}:=\left\{i\in H|\exists !k:i_{k}\neq h_{k}\right\}. \end{array}$$

The quantity *r*_***h***_ is obtained as the sum of the probabilities of multiple infections with only one haplotype ***i*** ∈ *U*_***h***_ and ***h***, or single infections with haplotype ***h***. An unambiguous observation with ***h*** and MOI *m* is obtained by randomly sampling *m*_***i***_ times ***i*** ∈ *U*_***h***_ and *m*_***h***_ = *m*−*m*_***i***_ times ***h***. Note, if *m*_***h***_ = *m* or *m*_***i***_ = *m*, a single infection with ***h*** or ***i*** is obtained, respectively. The latter are irrelevant for the prevalence of ***h***. One obtains


(19a) $$\begin{array}{l} \begin{array}{c} r_{h}:=\overset{\infty }{\underset{m=1}{\sum }}\kappa_{m}\underset{i\in U_{h}}{\sum }\overset{m-1}{\underset{m_{i}=1}{\sum }}\left(\begin{array}{l} m\\ m_{i} \end{array}\right)p_{i}^{m_{i}}p_{h}^{m-m_{i}}+\overset{\infty }{\underset{m=1}{\sum }}\kappa_{m}p_{h}^{m}. \end{array} \end{array}$$

As shown in Section Prevalence estimates of the Mathematical Appendix, this quantity simplifies to


(19b) $$\begin{array}{l} r_{h}=\underset{i\in U_{h}}{\sum }\left[G\left(p_{h}+p_{i}\right)-G\left(p_{i}\right)\right]-\left(n-1\right)G\left(p_{h}\right), \end{array}$$

where *G* is the PGF (1b).

The probability of all unambiguous observations $P\left(\tilde{O}\right)$ is derived in Section Prevalence estimates of the Mathematical Appendix and is given by


(20) $$\begin{array}{l} P\left(\tilde{O}\right)=\underset{h\in H}{\sum }\left[\frac{1}{2}\underset{i\in U_{h}}{\sum }[G\left(p_{h}+p_{i}\right)-G\left(p_{i}\right)]-\left(\frac{n}{2}-1\right)G\left(p_{h}\right)\right]. \end{array}$$

Hence, the prevalence of ***h*** conditioned on ambiguous observations is given by


(21) $$\begin{array}{l} r_{h|\tilde{O}}=\frac{\underset{i\in U_{h}}{\sum }\left[G\left(p_{h}+p_{i}\right)-G\left(p_{i}\right)\right]-\left(n-1\right)G\left(p_{h}\right)}{\underset{j\in H}{\sum }\left[\frac{1}{2}\underset{i\in U_{j}}{\sum }[G\left(p_{j}+p_{i}\right)-G\left(p_{i}\right)]-(\frac{n}{2}-1)G\left(p_{j}\right)\right]}. \end{array}$$

The conditional prevalence also exceeds the frequencies. Its value, however, seems to be closer to the frequencies *p*_***h***_ than that of the unobserved prevalence *q*_***h***_. The reason is that a mixed infection with haplotype ***h*** is much more likely to be ambiguous than unambiguous. Particularly, the fraction of single infections with haplotype ***h*** in unambiguous infections is disproportionately higher than in ambiguous infections. This is more pronounced if the genetic architecture of haplotypes consist of more loci (larger *n*). While this is true in theory, in real samples, when considering a large number of loci, unambiguous observations are increasingly unlikely (cf. **Figures 10**–**13**). The reason is that most haplotypes are not realized in a real population, which can be characterized by the presence of a few haplotypes which differ at multiple loci.

#### 3.1.3. Relative unambiguous prevalence

Due to unobservable information, a statistical model is required to obtain estimates for frequencies. However, in practice, “*ad-hoc*” estimates are popular if statistical methods are not available [cf. (50)]. Frequency estimates can be obtained, by first disregarding all ambiguous observations, calculate the empirically observed unambiguous prevalence of all haplotypes, and finally normalizing them - here we refer to this as the “relative unambiguous prevalence.” To assess how accurate such estimates are, this quantity can be expressed in terms of the statistical model introduced here, namely


(22a) $$\begin{array}{l} f_{h}:=\frac{r_{h|\tilde{O}}}{\underset{j\in H}{\sum }r_{j|\tilde{O}}}. \end{array}$$

Using (21) this can be rewritten as


(22b) $$\begin{array}{l} f_{h}=\frac{\underset{i\in U_{h}}{\sum }[G\left(p_{h}+p_{i}\right)-G\left(p_{i}\right)]-\left(n-1\right)G\left(p_{h}\right)}{\underset{j\in H}{\sum }\left[\underset{i\in U_{j}}{\sum }(G\left(p_{j}+p_{i}\right)-G\left(p_{i}\right))-\left(n-1\right)G\left(p_{j}\right)\right]}. \end{array}$$

Not surprisingly, the relative unambiguous prevalence resembles the frequency *p*_***h***_ of haplotpye ***h*** better than either the unobserved prevalence *q*_***h***_ or the unambiguous prevalence $r_{h|\tilde{O}}$. However, whether it is larger or smaller than the true frequency, *f*_***h***_ depends on the genetic architecture and the MOI distribution. In general there is no clear straightforward pattern, rendering the relative unambiguous prevalence an inadequate proxy for frequencies (cf. **Figures 10**–**13**).

### 3.2. Maximization of the likelihood function with the EM-algorithm

The maximum-likelihood (ML) method is employed here to obtain estimates for haplotype frequencies and the distribution of MOI. The likelihood function (11) derived in Section 2 does not permit a closed solution and has to be maximized numerically. A convenient and efficient method to maximize the likelihood function is the expectation maximization (EM)-algorithm. It is a two-step recursive method to find maximum likelihood estimates (MLEs). The steps of the EM-algorithm are: (i) the expectation (E) step (in which the expectation of the log-likelihood as a function of the unknown parameters conditioned on the parameter choice at the current iteration step is found), and (ii) the maximization (M) step (during which the function obtained at the E step is maximized with respect to the unknown parameters). The parameters obtained during the maximization step are then used in the next expectation step, and the algorithm is repeated until convergence. The algorithm is derived in the Mathematical Appendix in section Deriving the EM-algorithm.

In the present case the EM-algorithm leads to a two-step iterative procedure. The algorithm starts by choosing arbitrary initial values λ^(0)^ and ***p***^(0)^ for the Poisson parameter and haplotype frequencies. In step *t* + 1 the frequency estimates ***p***^(*t*+1)^ are derived as


(23a) $$\begin{array}{l} p_{h}^{\left(t+1\right)}=\frac{C_{h}^{\left(t\right)}}{\underset{h\in H}{\sum }C_{h}^{\left(t\right)}}, \end{array}$$

where


(23b) $$\begin{array}{l} C_{h}^{\left(t\right)}=p_{h}^{\left(t\right)}\underset{x\in O}{\sum }n_{x}\frac{\underset{y\in A_{x}}{\sum }{\left(-1\right)}^{N_{x}-N_{y}}G_{\lambda_{t}}^{\prime }\left(\underset{i\in A_{y}}{\sum }p_{i}^{\left(t\right)}\right)I_{A_{y}}\left(h\right)}{\underset{y\in A_{x}}{\sum }{\left(-1\right)}^{N_{x}-N_{y}}G_{\lambda_{t}}\left(\underset{i\in A_{y}}{\sum }p_{i}^{\left(t\right)}\right)}, \end{array}$$

(23c) $$I_{A_{y}}\left(h\right)=\{\begin{array}{ll} 1 & \text{if }h\in A_{y},\\ 0 & \text{if }h\notin A_{y}. \end{array}$$

and *G*_λ_*t*__(*z*) is the PGF (1b) with parameter λ_*t*_. The parameter λ_*t*+1_ is obtained by iterating the equation


(23d) $$\begin{array}{l} x_{\tau +1}=x_{\tau }-\frac{x_{\tau }-\frac{B_{t}}{N}\left(1-e^{-x_{\tau }}\right)}{1+x_{\tau }-\frac{x_{\tau }}{1-e^{-x_{\tau }}}}, \end{array}$$

where


(23e) $$\begin{array}{l} B_{t}=\underset{x\in O}{\sum }n_{x}\frac{\underset{y\in A_{x}}{\sum }{\left(-1\right)}^{N_{x}-N_{y}}\underset{h\in A_{y}}{\sum }p_{h}^{\left(t\right)}G_{\lambda_{t}}^{\prime }\left(\underset{i\in A_{y}}{\sum }p_{i}^{\left(t\right)}\right)}{\underset{y\in A_{x}}{\sum }{\left(-1\right)}^{N_{x}-N_{y}}G_{\lambda_{t}}\left(\underset{i\in A_{y}}{\sum }p_{i}^{\left(t\right)}\right)}, \end{array}$$

starting from *x*_0_ = λ_*t*_, until numerical convergence is reached. In particular, the iteration stops once |*x*_τ+1_ − *x*_τ_| < ε holds, by setting λ_*t*+1_ = *x*_τ+1_.

The EM-algorithm terminates once numerical convergence is reached. This is defined to be the case if $|\lambda_{t+1}-\lambda_{t}|+||p_{h}^{\left(t+1\right)}-p_{h}^{\left(t\right)}|{|}_{2}<\varepsilon$. The MLE are obtained as


(24) $$\begin{array}{l} {\hat{p}}_{h}=p_{h}^{\left(t+1\right)}\text{ and }\hat{\lambda }=\lambda_{t+1}. \end{array}$$

In practice, the EM-algorithm converges within a few iterations. Notably, it can be implemented efficiently. Because of the efficient implementation, bootstrap confidence intervals (CIs) can be readily obtained, as described in Confidence intervals.

An implementation of the EM-algorithm and the bootstrap CIs in R is available as Supplementary material. The code is also available at: https://github.com/Maths-against-Malaria/MultiLociBiallelicModel.git.

### 3.3. Using a plug-in estimate for the Poisson parameter

Estimates of MOI might be unreliable in the case of unbalanced haplotype frequency distributions, as they typically occur for drug-resistance associated haplotypes in malaria. To compensate for this, the number of loci considered can be increased. In the case of resistance-associated haplotypes a typical choice would be a set of unlinked neutral marker loci, which are likely to have balanced frequencies.

However, adding additional loci can lead to three problems in practice. First, due to the curse of dimensionality, the number of parameters to be estimated becomes so large that haplotypes are characterized at the individual level. This can be compensated by marginalizing the frequency estimates with regard to the set of loci of interest. Second, poor data quality can lead to missing data entries, so that the number of samples, which have information at all loci (the original and the additional set), is substantially smaller than if the sets of loci would be considered separately. Similarly, one set of loci might just have been process for a sub-sample. Third, molecular information might, for both sets of loci, have been performed for different sets of samples. In any of these cases, one could estimate the Poisson parameter λ based only on the additional set of loci and use this estimate as a plug-in estimate to obtain the haplotype frequency distribution for the original set of loci.

If one prefers to use a plug-in estimate for the Poisson parameter λ, the EM-algorithm can be adapted. This adaptation is derived in The EM-algorithm using a plug-in estimate for the Poisson parameter. This algorithm is also implemented as an R script (see the available User manual in the Supplementary material).

Notably, it is advisable to use the whole available data, rather than plug-in estimates, unless one of the situations outlined above applies. The reason is that as a general guideline, estimates should be based if possible on the full information being available.

### 3.4. Estimating samplewise MOI

Once the population-level MOI parameter λ and the haplotype frequencies ***p*** have been estimated as $\hat{\lambda }$ and $\hat{p}$, these can be used as plug-in estimates to infer the true MOI *m* for a sample ***x***. In line with maximum-likelihood estimation, a natural estimate $\hat{m}$ is the value of *m* which maximizes that has the highest probability given the observation ***x*** and the plug-in estimates $\hat{\lambda }$ and $\hat{p}$. More precisely, the samplewise estimate $\hat{m}$ of MOI is


(25) $$\begin{array}{l} \hat{m}=\underset{m}{arg max}P\left(\text{MOI}=m|x;\hat{\lambda },\hat{p}\right), \end{array}$$

where $P\left(\text{MOI}=m|x;\hat{\lambda },\hat{p}\right)$ is defined in Samplewise MOI of the Mathematical Appendix. The samplewise MOI estimates are implemented in the R script available at https://github.com/Maths-against-Malaria/MultiLociBiallelicModel.git. A numerical example is found in the User manual.

### 3.5. Data application

As an application, we estimated MOI and haplotype frequencies of malaria parasites associated with resistance to sulfadoxine-pyrimethamine (SP) in Cameroon in 2001/2002 and 2004/2005. MOI was estimated to be intermediate at both time points, and slightly decreased in 2004/2005. The estimated MOI parameters were $\hat{\lambda }=0.9397$ (95% CI: 0.7492, 1.1551) for 2001/2002 and $\hat{\lambda }=0.8645$ (95% CI: 0.6496, 1.0928) for 2004/2005. This slight decrease is in accordance with the downward trend in the number of reported cases in Cameroon from 1992 to 2005, sustained by programs like the “Roll Back Malaria” program [cf. (51)].

The estimates of haplotype frequencies are presented in Table 3 (confidence intervals for the frequencies estimates are omitted to improve readability). The drug sensitive wildtype and those with single mutations decreased in frequency between the two time points, whereas strongly resistant haplotypes with triple mutations on *dhfr* and double mutations at *dhps* increased in frequency. This is not surprising considering that SP drug pressure was high during that time. Namely, chloroquine was officially removed as first line therapy in Cameroon in 2002, whereas amodiaquine and SP became first- and second-line treatments [cf. (42)]. Although, the combination of artesunate and amodiaquine became the official therapy for uncomplicated *P. falciparum* malaria in 2004, it was not widely used until 2007 [cf. (42)]. In particular, the frequency of the highly resistant haplotypes 51**I**/59**R**/108**N**/I164—S436/437**G**/K540/A581/A613 increased from 38 (95% CI: 31.27, 44.37%) to 46% (95% CI: 39.28, 53.44%) (see Table 3), whereas the less resistant haplotypes characterized by just two mutations in *dhfr* decreased in frequency.

**Table 3:** Frequencies estimates of SP-resistant haplotypes from the Cameroonian data.

**Haplotype**		**Frequency ***p*****		**Prevalence ***p*****		**Cond. prevalence ***p*****		***Ad-hoc*** **freq**. ***p***	
		**Years**		**Years**		**Years**		**Years**	
***dhfr***	***dhps***	**01/02**	**04/05**	**01/02**	**04/05**	**01/02**	**04/05**	**01/02**	**04/05**
NCSI	SAKAA	0.0190	0.00468	0.0290	0.0070	0.0168	0.0037	0.0204	0.0063
NCSI	**A**AKAA	0.0604	0.00548	0.0905	0.0082	0.0521	0.0043	0.0476	0.0063
NCSI	S**G**KAA	0.0178	0.00965	0.0272	0.0144	0.0151	0.0075	0.0136	0.0063
**I**C**N**I	S**G**KAA	0.0164	< 10^−12^	0.0250	< 10^−12^	0.0194	< 10^−12^	0.0272	−−
N**RN**I	SAKAA	0.0095	0.01025	0.0145	0.0152	0.0084	0.0084	0.0136	0.0127
N**RN**I	**A**AKAA	0.0122	< 10^−12^	0.0188	< 10^−12^	0.0130	< 10^−12^	0.0136	−−
N**RN**I	S**G**KAA	0.0475	0.02179	0.0717	0.0322	0.0583	0.0257	0.0612	0.0253
N**RN**I	**AG**KAA	0.0134	0.00935	0.0205	0.0139	0.0124	0.0082	0.0204	0.0127
**IRN**I	SAKAA	0.0271	0.04085	0.0413	0.0600	0.0387	0.0564	0.0340	0.0506
**IRN**I	**A**AKAA	0.2447	0.25216	0.3372	0.3384	0.2493	0.2515	0.1973	0.2089
**IRN**I	S**G**KAA	0.3792	0.46288	0.4921	0.5698	0.4325	0.5202	0.3741	0.4494
**IRN**I	SAKA**T**	0.0265	0.00776	0.0404	0.0116	0.0230	0.0063	0.0204	0.0063
**IRN**I	**AG**KAA	0.0606	0.12062	0.0909	0.1711	0.0867	0.1714	0.0884	0.1519
**IRN**I	S**G**KA**T**	0.0165	< 10^−12^	0.0252	< 10^−12^	0.0198	< 10^−12^	0.0204	−−

The first column shows the amino acid sequence at codons 51, 59, 108, and 164 at the *dhfr* locus and the second column shows the amino acid sequence at codons 436, 437, 540, 581, and 613 at the *dhps* locus. A total of 50 and 36 haplotypes were estimated to have strictly positive frequency in 2001/2002 and 2004/2005, respectively. Only haplotypes with an estimated frequency >0.01 in 2001/2002 or 2004/2005 are reported. The remaining columns show the MLE of the respective frequencies, the estimated prevalence, the estimated conditional prevalence, and the *ad-hoc* estimate (relative unambiguous prevalence) for the haplotype frequencies. Cond., Conditional; Freq., Frequency.

Although the *ad-hoc* frequency estimates given by the relative unambiguous prevalence (see above) are close to the MLE, some frequency estimates differ substantially. For instance, the MLEs for the frequency of the highly resistant haplotype 51**I**/59**R**/108**N**/I164—436**A**/A437/K540/A581/A613 at both time points are, respectively, 24.5% (95% CI: 18.98, 30.41%) and 25.2% (95% CI: 19.53, 30.93%), the corresponding *ad-hoc* estimates are 19.7 and 20.9% (see Table 3). This is not surprising because, this haplotype is likely to occur in mixed infections with the predominant haplotype, which would be disregarded by the *ad-hoc* estimates.

Obvious is the difference between frequency and prevalence estimates. The frequencies of the highly resistant haplotype 51**I**/59**R**/108**N**/I164—S436/437**G**/K540/A581/A613 at both time points—38 and 46%—are substantially lower than the prevalences, estimated to be 49 and 57%, respectively (see Table 3). However, the estimates for the conditional prevalence (which are not recommendable) are only slightly smaller than the prevalence estimates and amount to 43 and 52%. Since MOI is intermediate, the discrepancy is not expected to be too large.

The same is true for the other highly resistant haplotype 51**I**/59**R**/108**N**/I164—436**A**/A437/K540/A581/ A613. The frequency estimates are 24.5% (95% CI: 18.59, 30.07%) and 25.2% (95% CI: 18.84, 30.95%), whereas the prevalence estimates are 33.7 and 33.8%. Although MOI is intermediate, the discrepancy between the prevalence and conditional prevalence estimates (24.9 and 25.2% at the two time points) are quite large (cf. Table 3).

### 3.6. Performance of the estimator

Ideally an estimator is (i) unbiased, i.e., it is accurate and (ii) precise, i.e., it has low variance. The minimal variance of an unbiased estimator is given by the Cramér-Rao lower bound. Typically, MLEs have good asymptotic properties. They are asymptotically unbiased and efficient (they asymptotically attain the minimal possible variance). Despite these desirable asymptotic properties, the quality of MLEs has to be investigated under finite sample sizes. If bias is small and the variance of the estimator is close to the Cramér-Rao lower bound, one has confidence that the estimator is “optimal.” The quality of an estimator is measured by the mean squared error (MSE)


(26) $$\begin{array}{l} \text{MSE}\left(\hat{\theta }\right)=\text{Var}\left(\hat{\theta }\right)+{\left(\text{Bias}\left(\hat{\theta }\right)\right)}^{2}, \end{array}$$

which is the sum of the estimators variance and squared bias.

Since, the MLE here has no closed solution, bias and variance cannot be studied analytically. We therefore perform a numerical simulation to study the estimator's bias and variance.

#### 3.6.1. Bias of the estimator

To compare bias across a range of different parameter values, we consider a ‘dimensionless’ quantity, namely the relative bias (14a) in percent, i.e., the bias of the estimator in percent of the true value of the parameter.

##### 3.6.1.1. Relative bias for haplotypes frequencies estimates

**Figure 2:**
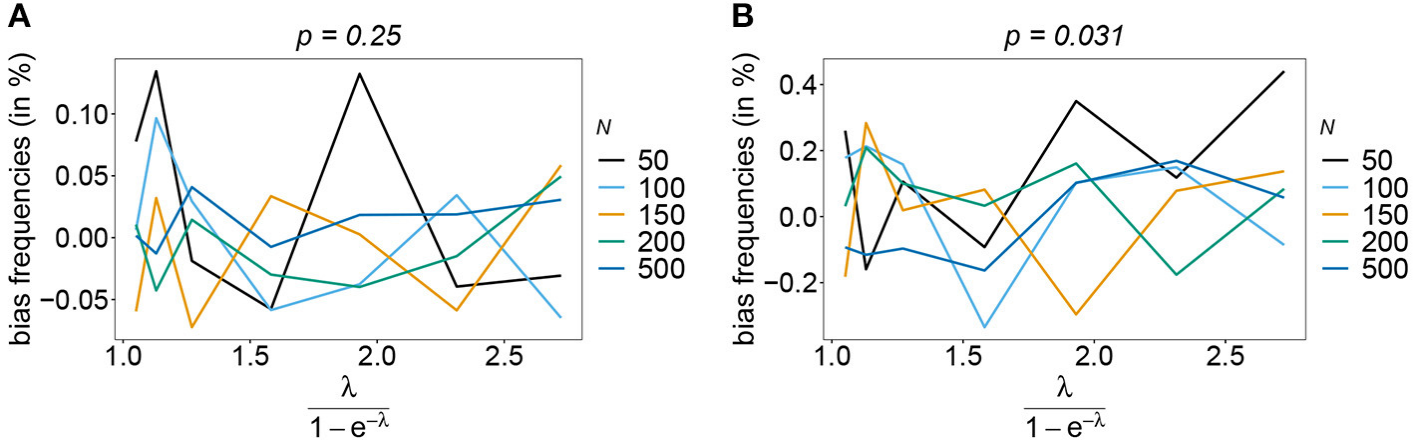
Bias of frequencies estimates—the symmetric case. Shown is the bias of the frequency estimates in % as a function of the mean MOI (i.e., for a range of Poisson parameters). The symmetric haplotype frequency distributions (cf. Table 1) for *n* = 2 **(A)** and *n* = 5 **(B)** are assumed. In both panels, only the bias for the first haplotype is shown (in the symmetric case, all haplotypes are equivalent and bias looks similarly). Colors correspond to different sample sizes.

The estimator is typically unbiased, with no noticeable effect of sample size if the true haplotype distribution is symmetric independently of the number of considered loci *n*, i.e., all haplotypes are equally abundant in the pathogen population (see Figure 2). This is intuitively expected, because the haplotypes are interchangeable in this case. Deviations from the true frequency distribution occur only due to random sampling. Although random effects are more pronounced for small sample size and larger *n* (also seen from the variation in Figure 2), in terms of bias this effect averages out. However, it will affect the MLE's variance (see below).

**Figure 3:**
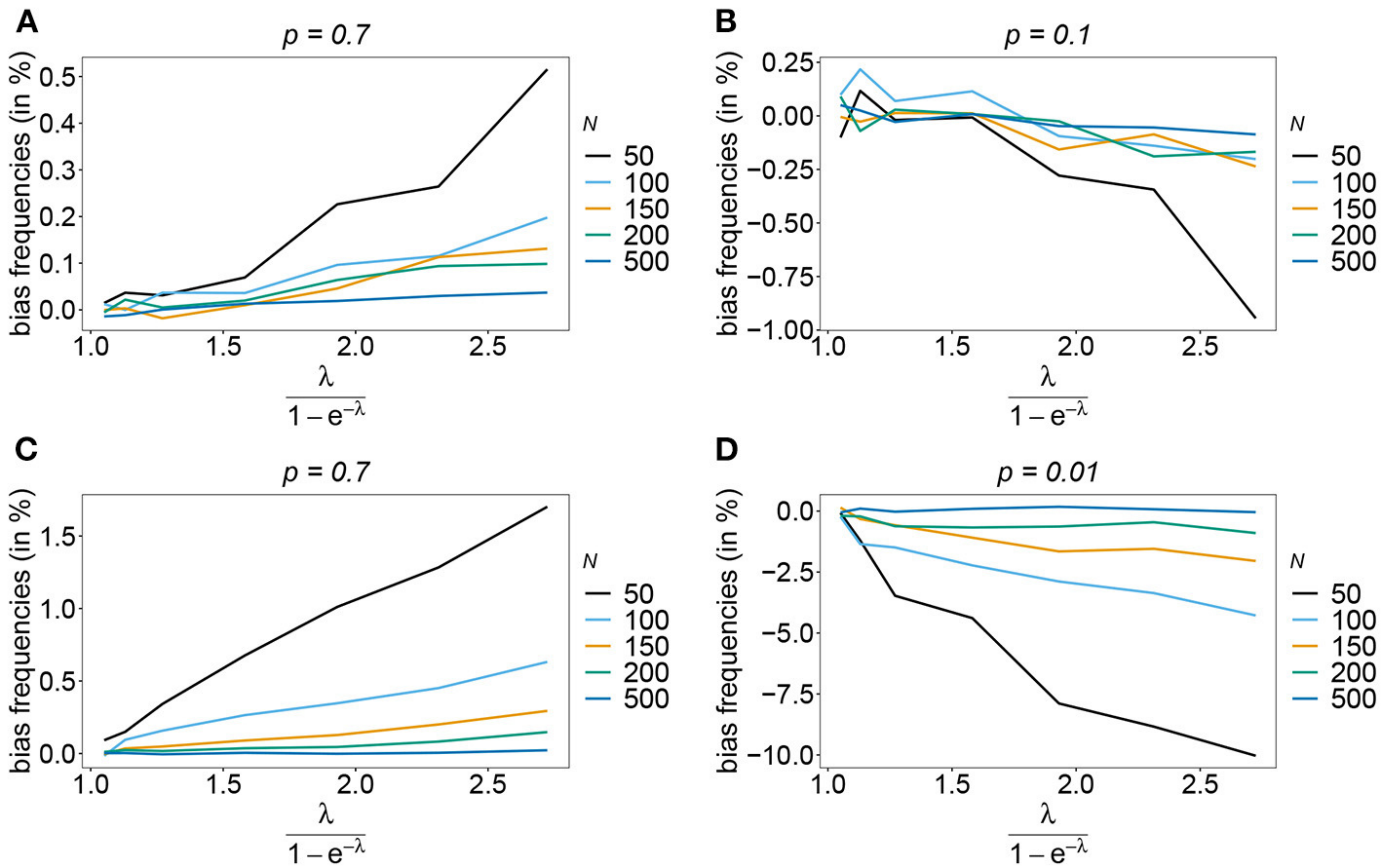
Bias of frequencies estimates—the unbalanced case. Shown is the bias of the frequency estimates in % as a function of the mean MOI (i.e., for a range of Poisson parameters). The skewed haplotype frequency distributions (cf. Table 1) for *n* = 2 **(A,B)** and *n* = 5 **(C,D)** are assumed. In both cases only the bias for the predominant haplotype and one underrepresented haplotype are shown (all underrepresented haplotypes are equivalent and bias looks similarly). Colors correspond to different sample sizes.

Also, if the underlying haplotype frequency distribution is skewed, the estimator has low bias (see Figure 3). Bias (in relative terms) is highest for haplotypes with low frequencies. These tend to be underrepresented in datasets. On the contrary, the frequencies of predominant haplotypes will be overestimated, as these tend to be over-represented in datasets. This is particularly true for high MOI (ψ > 1.8) and small sample size. Bias vanishes with increasing sample size. The estimates can be considered almost unbiased for *N* ≥ 150. Note that the bias of rare haplotypes will only be large in relative terms, not in absolute terms. In practice, large sample size is required to detect rare haplotypes. Bias tends to be larger, if a larger number of loci is considered (compare Figures 3A,B with Figures 3C,D). The reason is that the number of possible haplotypes is increasing geometrically with larger *n* and in the numerical examples a large number of haplotypes with low frequencies are assumed.

**Figure 4:**
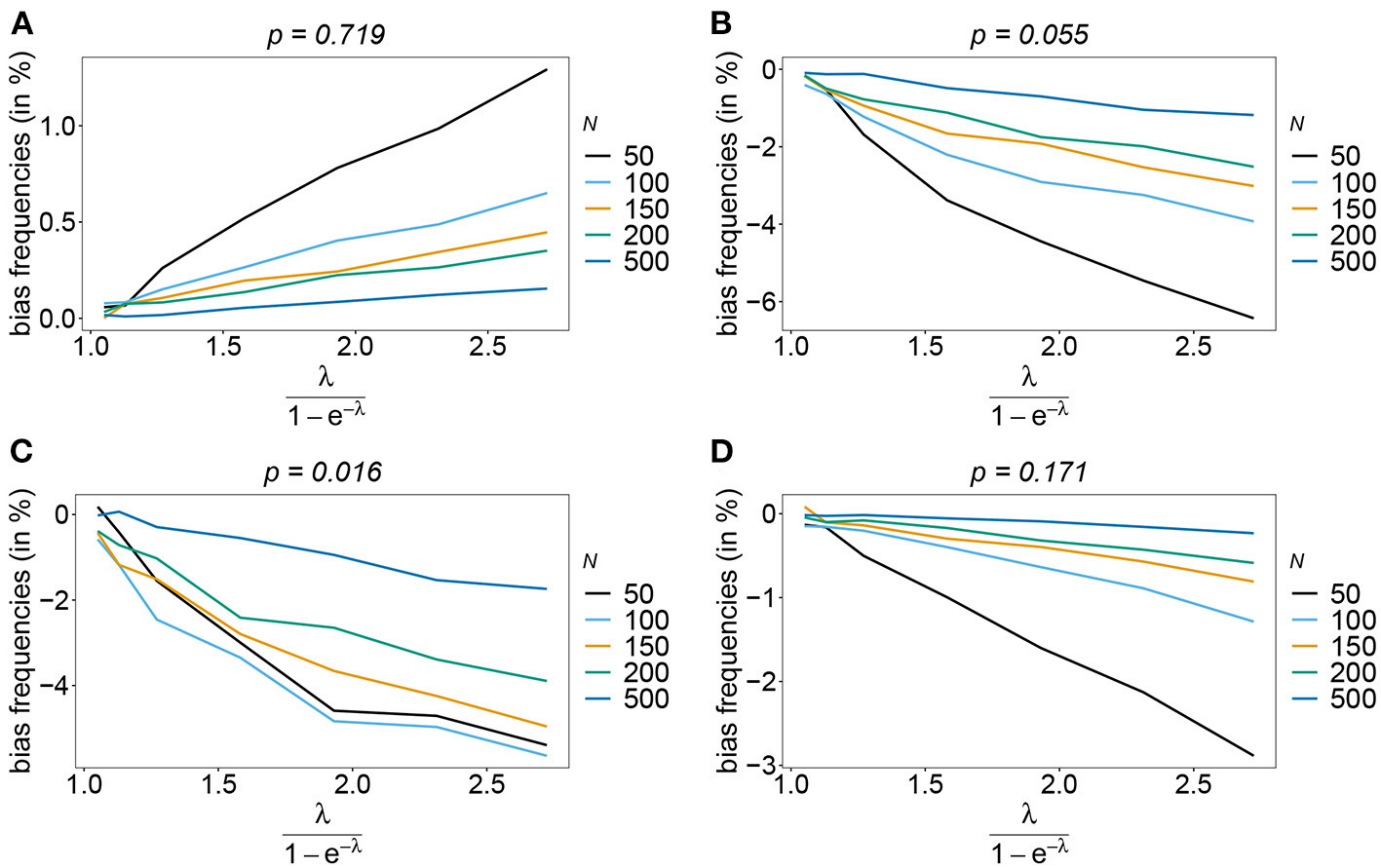
Bias of frequencies estimates—Kenya data 2005. Shown is the bias of the frequency estimates in % as a function of the mean MOI (i.e., for a range of Poisson parameters). The haplotype frequency distributions (cf. Table 2) for *n* = 10 are assumed. The bias for the predominant haplotype and few underrepresented haplotype are shown **(A–D)**, the corresponding haplotype frequencies are shown at the top of the panels. Colors correspond to different sample sizes.

**Figure 5:**
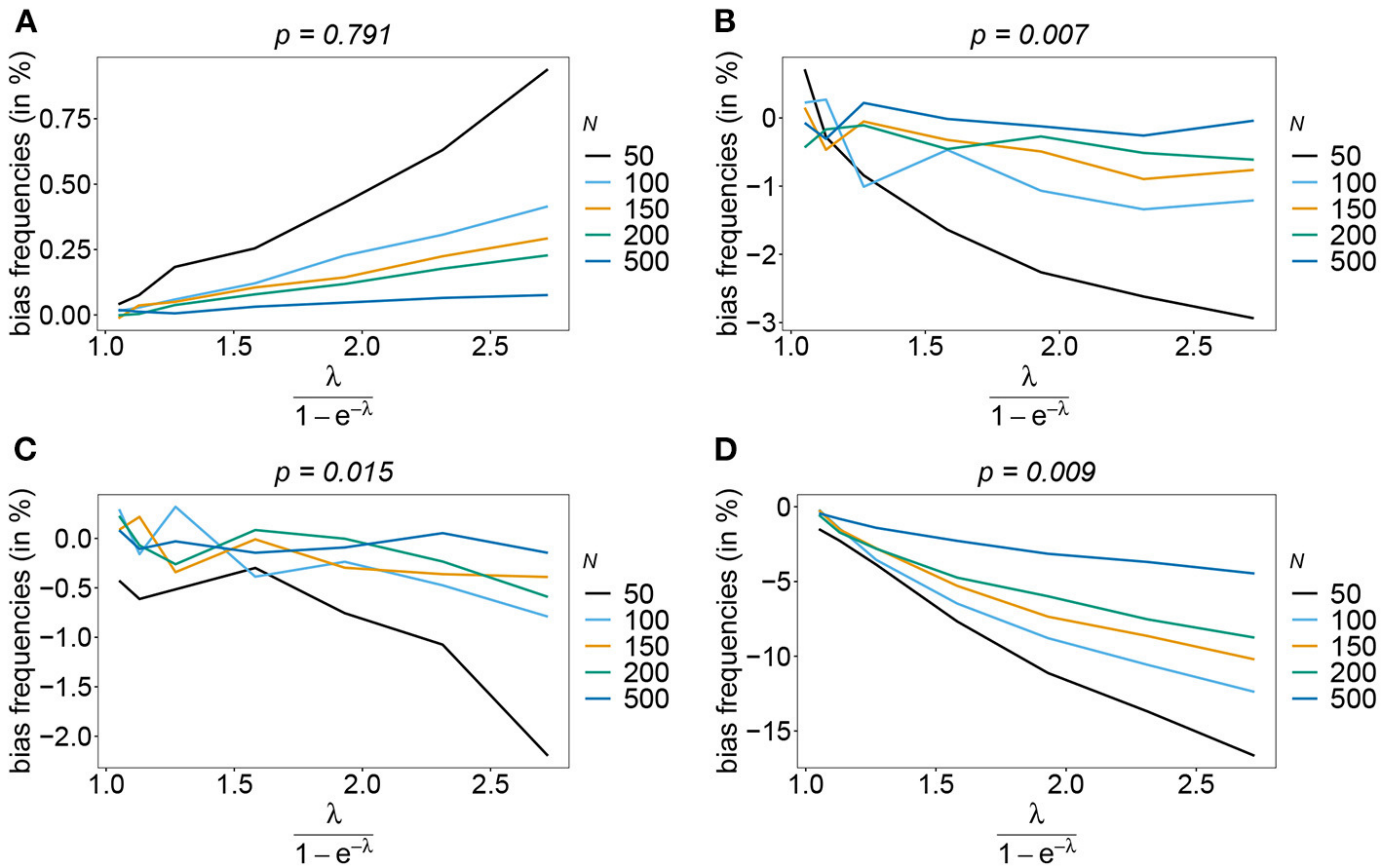
Bias of frequencies estimates—Kenya data 2010. Shown is the bias of the frequency estimates in % as a function of the mean MOI (i.e., for a range of Poisson parameters). The haplotype frequency distributions (cf. Table 2) for *n* = 10 are assumed. The bias for the predominant haplotype and few underrepresented haplotype are shown **(A–D)**, the corresponding haplotype frequencies are shown at the top of the panels. Colors correspond to different sample sizes.

The number of possible haplotypes increases geometrically with the number of considered loci. For *n* = 10, 1,024 possible haplotypes exist. In practice only a fraction of the possible haplotypes circulate in the population. Figures 4, 5 show the bias of haplotype frequencies assuming the frequency distributions of malaria haplotypes estimated in Kenya. These are two rather unbalanced frequency distributions. Also in these cases, the frequency estimates have little bias that vanishes with increasing sample size. Again the frequencies of predominant haplotypes tend to be overestimated, while those of rare haplotypes tend to be underestimated. Bias increases with increasing MOI. The reason is that super-infections are common, and rare haplotypes will be unlikely to occur in single infections. However, they will likely occur together in mixed infections with predominant types, resulting in ambiguous information. As a consequence, the estimator yields positive frequency estimates for haplotypes that are not circulating in the population, and thereby understimates those rare haplotype that are actually present. (Note that in practice, due to ambiguity, it is typically impossible to determine which haplotypes are actually circulating in the population.)

##### 3.6.1.2. Relative bias for MOI parameter estimates

**Figure 6:**
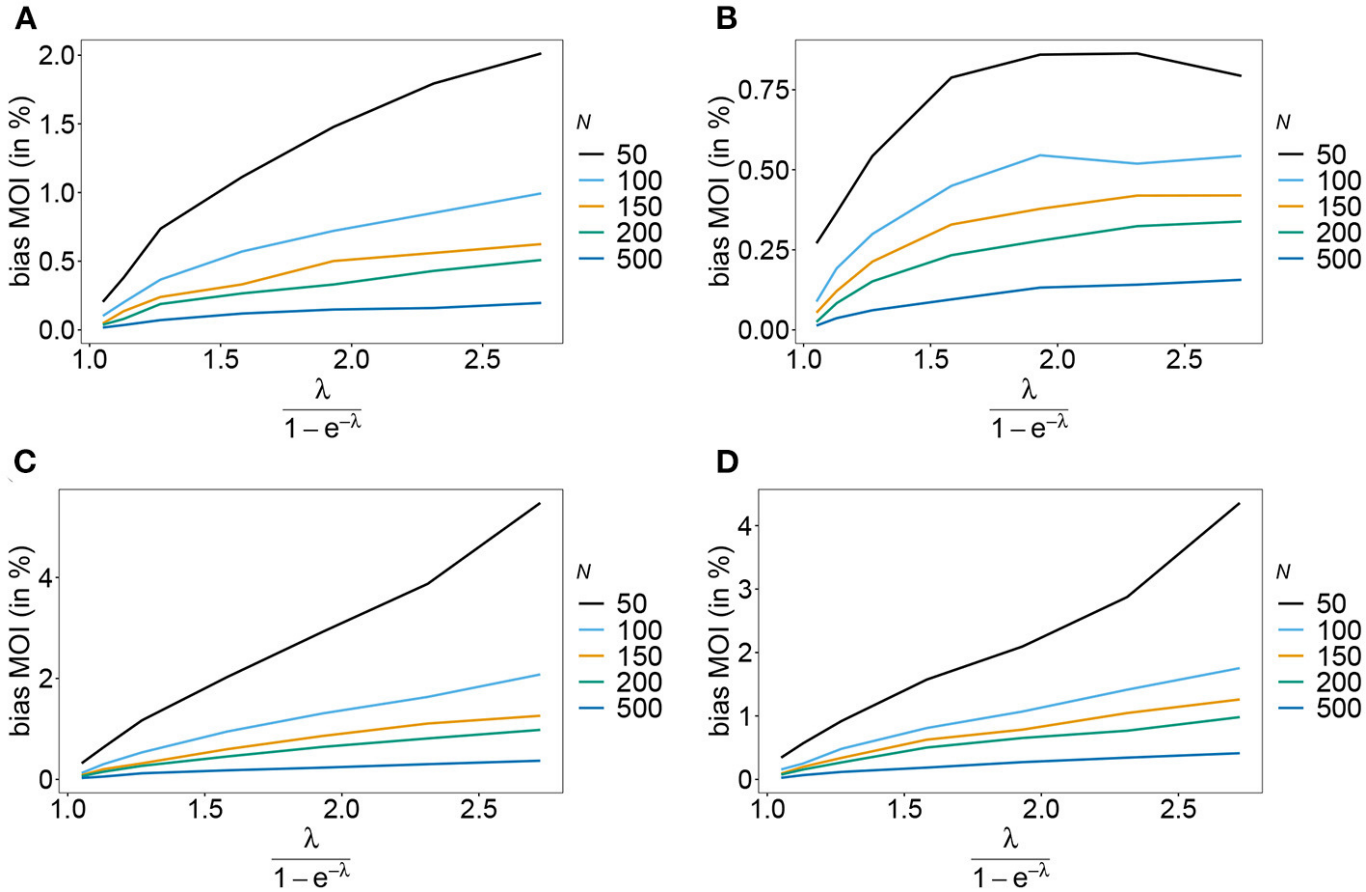
Bias of MOI estimates. Shown is the bias of the mean MOI estimates ψ in % as a function of the true mean MOI (i.e., for a range of Poisson parameters). Symmetric haplotype frequency distributions (Table 1) are assumed for *n* = 2 **(A)** and *n* = 5 **(B)**, whereas skewed distributions are assumed in **(C,D)**, for *n* = 2 and *n* = 5, respectively. Colors correspond to different sample sizes.

**Figure 7:**
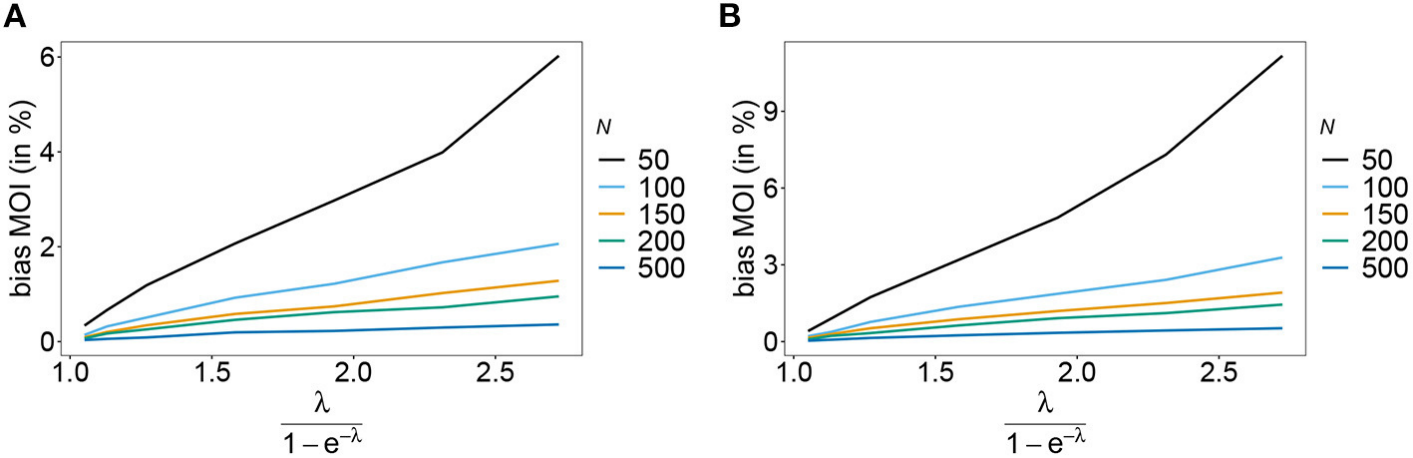
Bias of MOI estimates. As in Figure 6 but for *n* = 10 for the haplotype estimated for antimalarial drug resistance in Kenya (see Table 2) in 2005 **(A)** and 2010 **(B)**.

Rather than evaluating the bias of the MOI parameter λ, bias is evaluated in term of the empirically more relevant mean MOI ψ. For a given *n*, the estimator has relatively little bias irrespective of the frequency distribution and true value of λ. In general, the estimator overestimates the true parameter. The reason is that λ is positive, and can be overestimated but not underestimated by arbitrary amounts. In general MLEs are sensitive to outliers. Here, particularly for large λ, rare over-representations of multiple infections in the data, lead to substantial overestimates. This is more likely to occur for small sample sizes and large *n*. Consequently, bias is increasing as a function of λ and decreasing as a function of sample size *N*. Typically, bias is decreasing for larger *n*, because the amount of information contained in a dataset increases. However, also sample size has to be adequate for larger *n* to appropriately represent the haplotype distribution. Not surprisingly, bias is higher for more skewed frequency distributions. This is because, rare haplotypes will be underrepresented and single infections with rare haplotypes are unlikely to be observed in a dataset, particularly for large ψ (see Figures 6, 7). In general, bias is small for samples of size *N*≥ 150.

#### 3.6.2. Variance of the estimator

The estimator's variance was assessed in terms of the coefficient of variation (14b). This is a dimensionless quantity, which allows comparisons across a range of parameter values.

**Figure 8:**
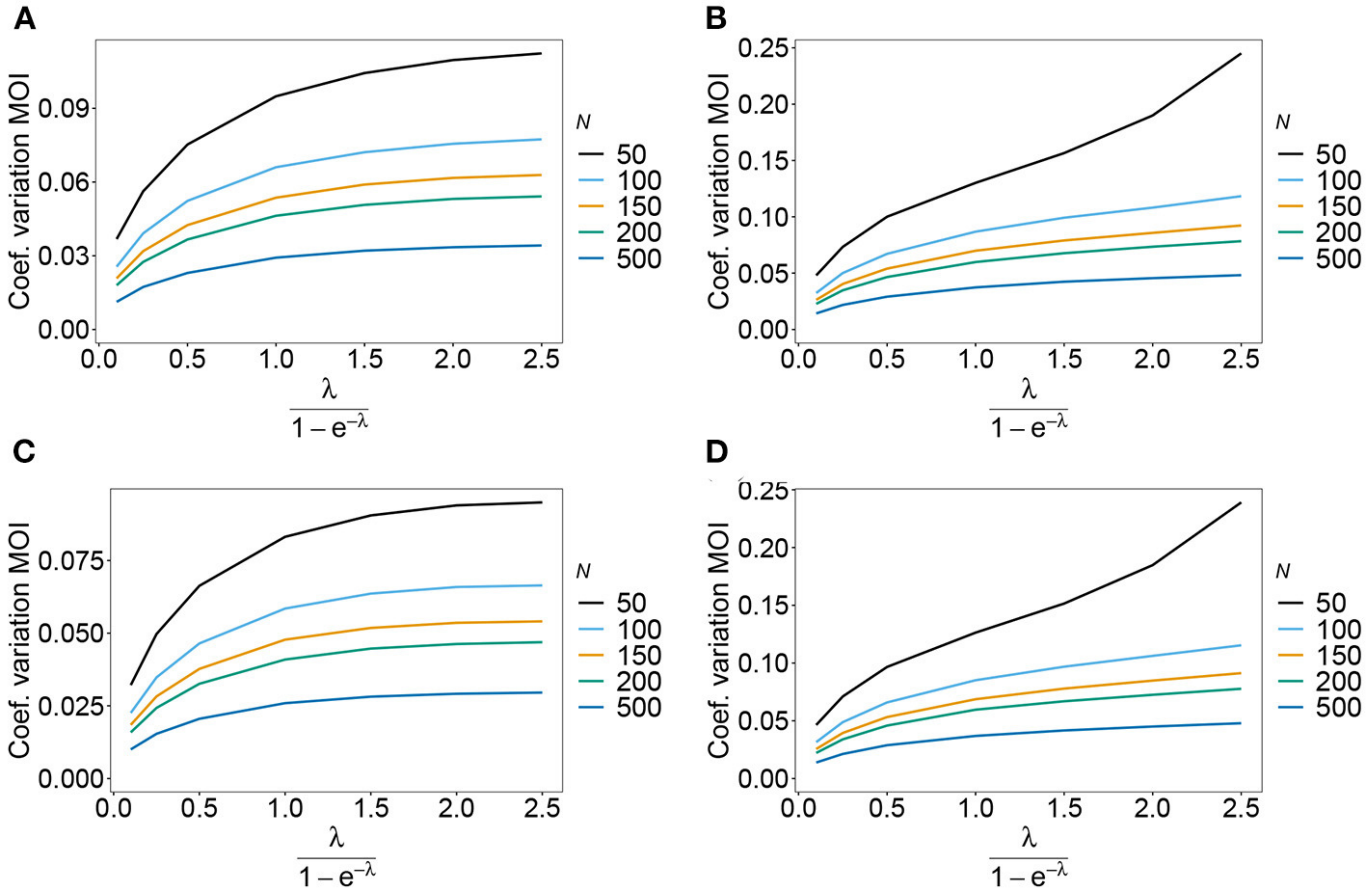
Variance of MOI estimates. Shown is the variance of the mean MOI estimates ψ in % as a function of the true mean MOI (i.e., for a range of Poisson parameters). The symmetric haplotype frequency distribution is assumed respectively for *n* = 2 and *n* = 5 **(A,B)** as well as the skewed haplotype frequency distributions **(C,D)** (cf. Table 1). Colors correspond to different sample sizes.

**Figure 9:**
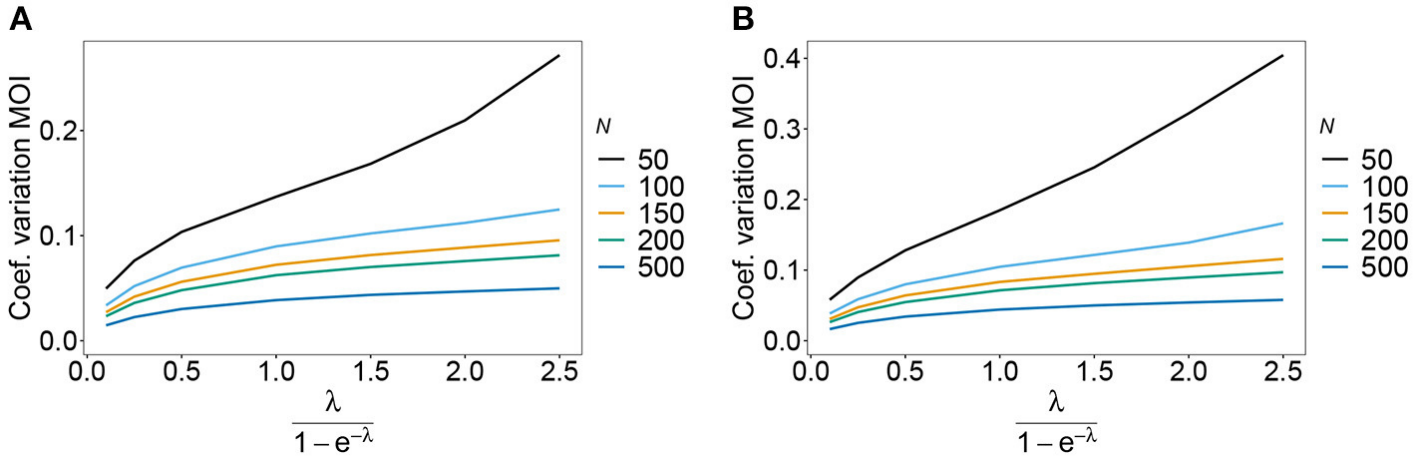
Variance of MOI estimates. Shown is the variance of the mean MOI estimates ψ in % as a function of the true mean MOI (i.e., for a range of Poisson parameters). The haplotype frequency distributions for *n* = 10 are assumed, respectively, for the year 2005 **(A)** and 2010 **(B)** (cf. Table 2). Colors correspond to different sample sizes.

As expected the estimator's variance decreases with increasing sample size *N*, because datasets reflect the underlying population more accurately, leading to less fluctuations between different realizations of datasets. The variance of the MOI estimator is relatively small (see Figures 8, 9). Not surprisingly, it increases with *n* (compare Figures 8A,C with Figures 8B,D and see Figure 9). The number of haplotypes increases with *n*, and hence, less information is available for each single haplotype. Thus, for larger *n*, due to the curse of dimensionality, the underlying population is less adequately represented in a dataset of given sample size *N*.

The variance of the frequency estimates has similar properties (not shown).

#### 3.6.3. Prevalence and relative prevalence

**Figure 10:**
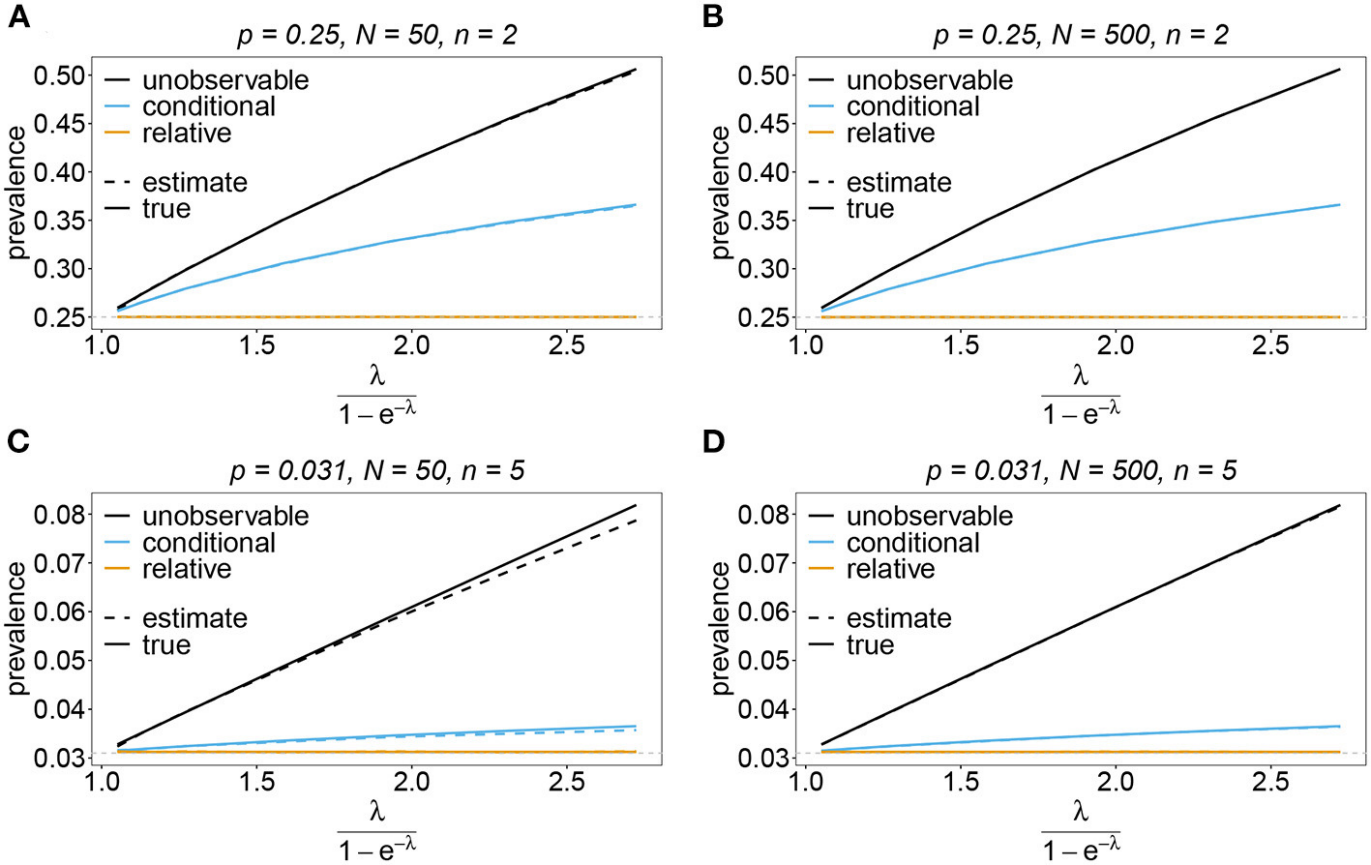
Prevalence estimates—the symmetric case. Shown is the prevalence of the haplotypes as a function of the mean MOI (i.e., for a range of Poisson parameters). The symmetric haplotype frequency distributions (cf. Table 1) for *n* = 2 **(A,B)** and *n* = 5 **(C,D)** are assumed. In both cases of *n* only the prevalence estimates for the first haplotype are shown for a small (*N* = 50) and big (*N* = 500) sample size (in the symmetric case all haplotypes are equivalent and prevalence looks similarly). Colors correspond to different prevalence models. The solid line show the true prevalence and the dashed line the estimates.

**Figure 11:**
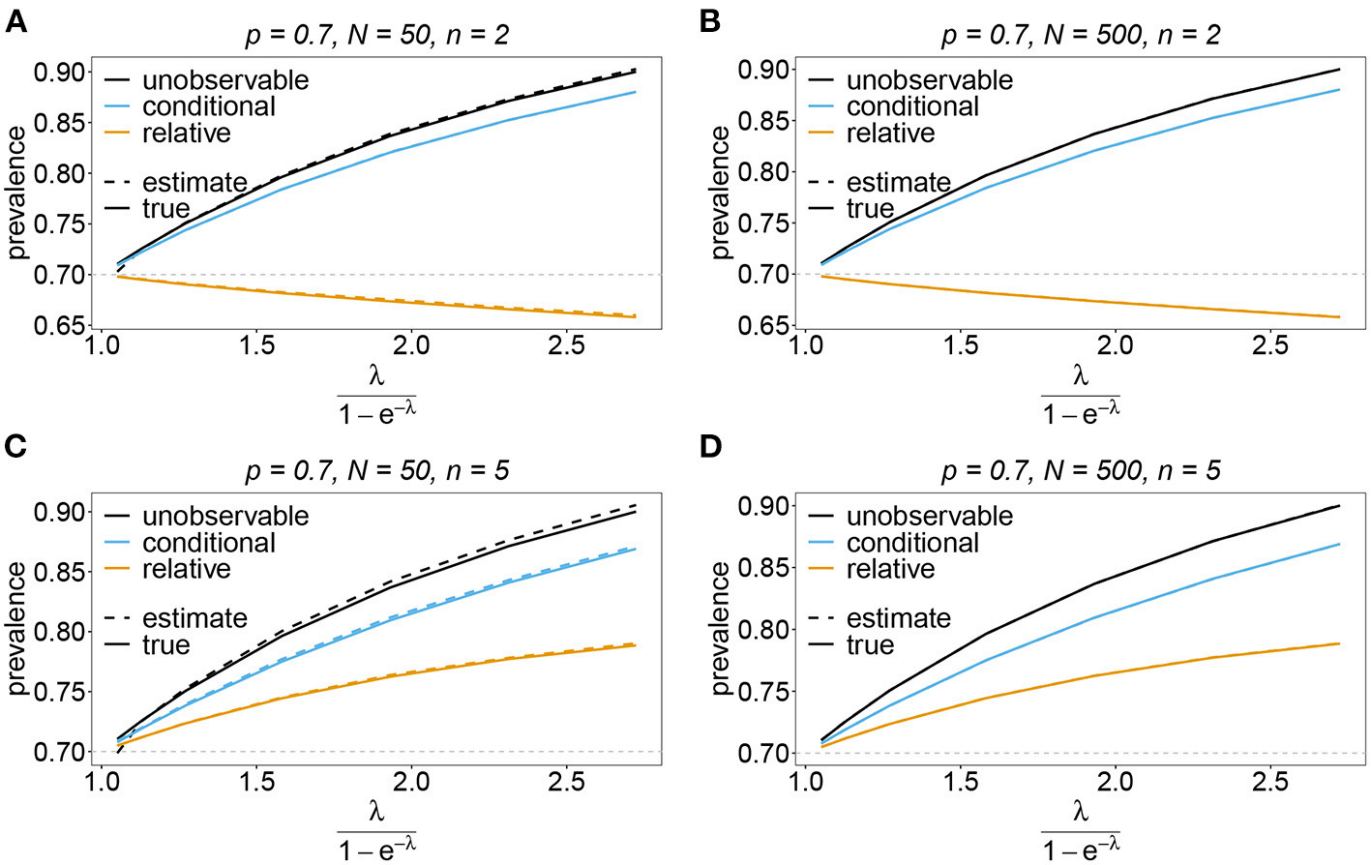
Prevalence estimates—the unbalanced case. Shown is the prevalence of the predominant haplotypes as a function of the mean MOI (i.e., for a range of Poisson parameters). The skewed haplotype frequency distributions (cf. Table 1) for *n* = 2 **(A,B)** and *n* = 5 **(C,D)** are assumed. In both cases of *n* only the prevalence estimates are shown for a small (*N* = 50) and big (*N* = 500) sample size. Colors correspond to different prevalence models. The solid lines show the true prevalence and the dashed lines the estimates.

**Figure 12:**
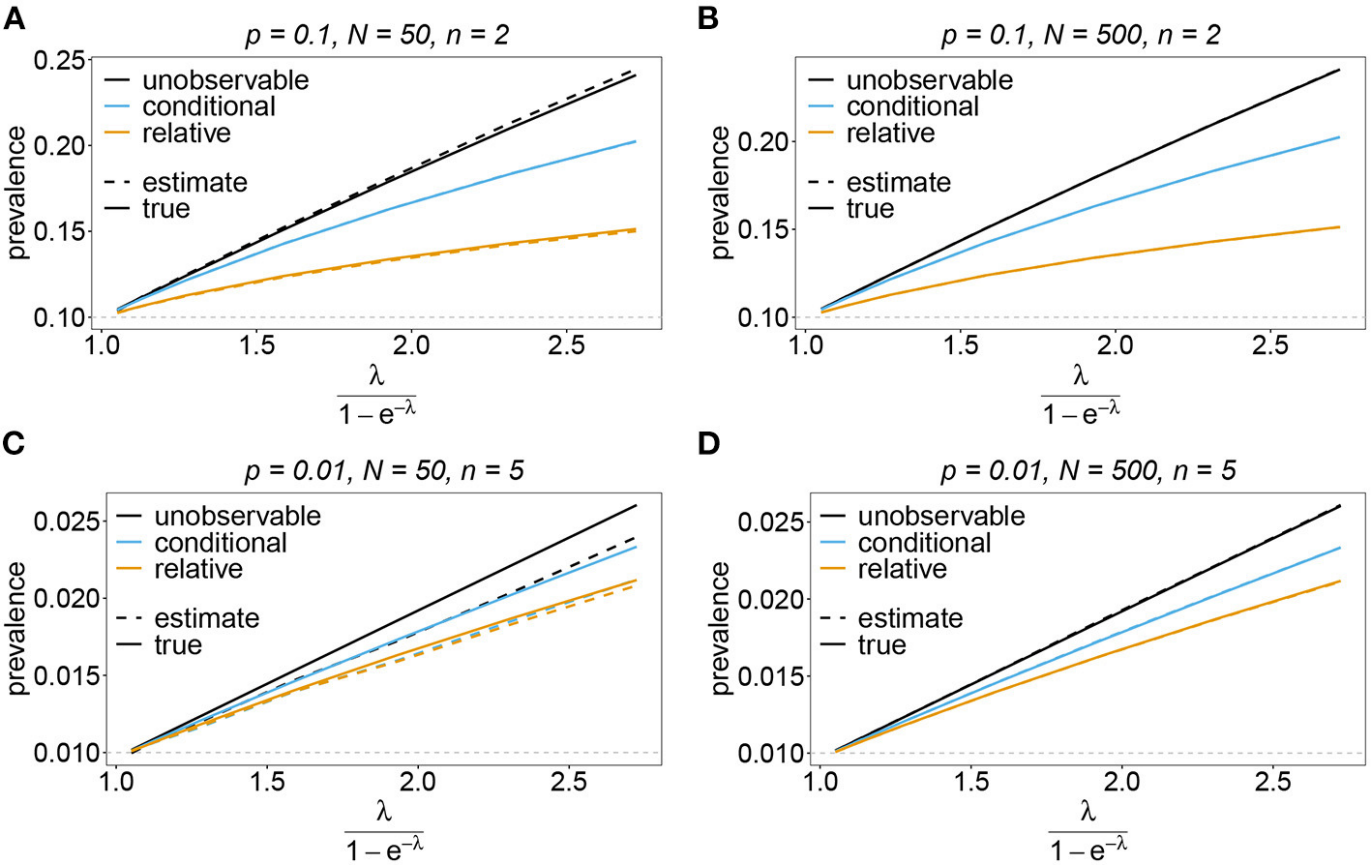
Prevalence estimates—the unbalanced case: Shown is the prevalence of the underrepresented haplotypes as a function of the mean MOI (i.e., for a range of Poisson parameters). The skewed haplotype frequency distributions (cf. Table 1) for *n* = 2 **(A,B)** and *n* = 5 **(C,D)** are assumed. In both cases of *n* only the prevalence of one of the underrepresented haplotypes estimates are shown for a small (*N* = 50) and big (*N* = 500) sample size (all underrepresented haplotypes are equivalent and prevalence looks similarly). Colors correspond to different prevalence models. The solid lines show the true prevalence and the dashed lines the estimates.

**Figure 13:**
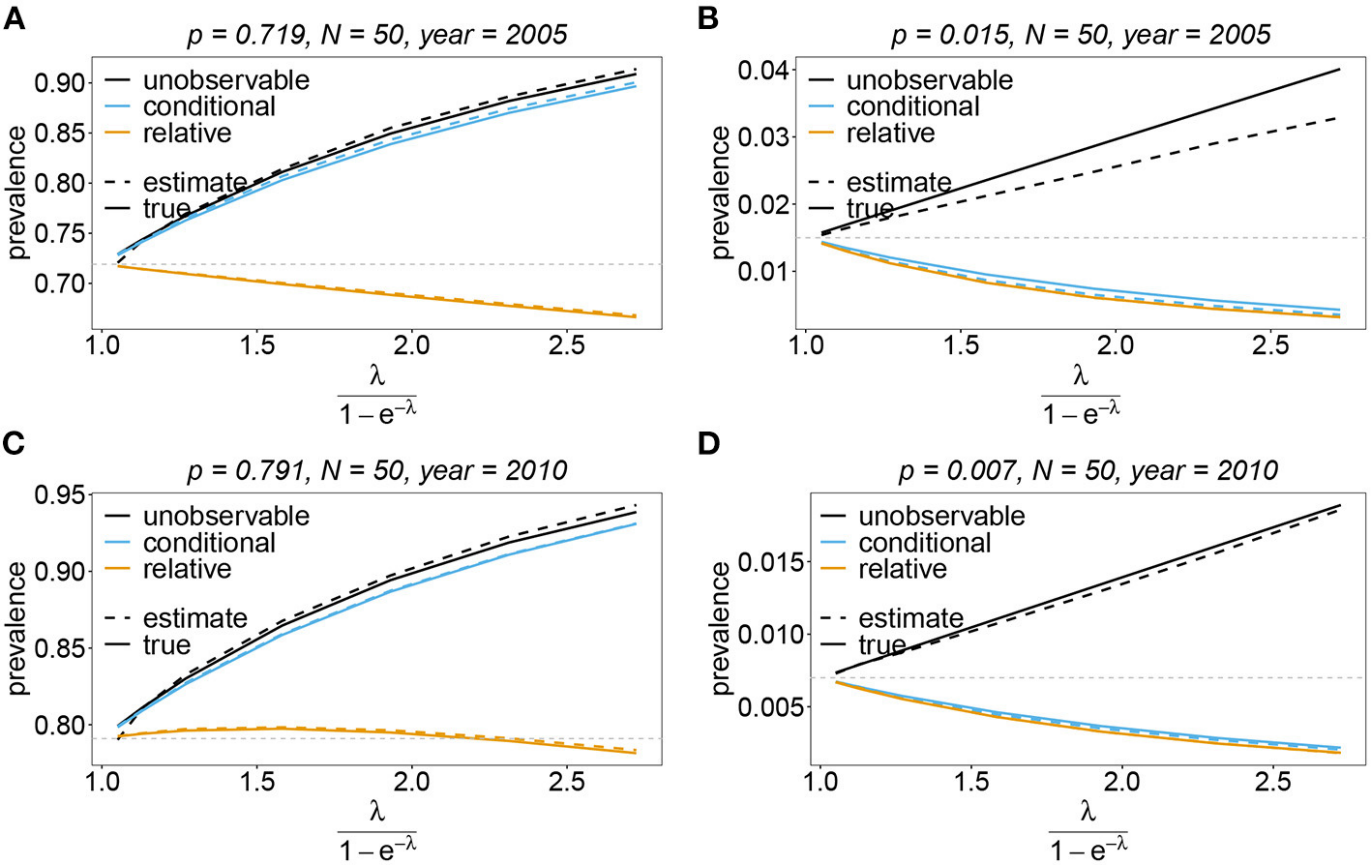
Prevalence estimates—the unbalanced case: Shown is the prevalence of the dominant and one underrepresented haplotype for the years 2005 **(A,B)** and 2010 **(C,D)** as a function of the mean MOI (i.e., for a range of Poisson parameters). The haplotype frequency distributions (cf. Table 2) for *n* = 10 are assumed. The prevalence estimates are shown for *N* = 50. Colors correspond to different prevalence models. The solid lines show the true prevalence and the dashed lines the estimates.

Sometimes the prevalence of haplotypes is empirically more important than their frequencies. For example, in the case of drug resistance, clinically it is more relevant to assess the probability that a patient is infected with a resistant haplotype, rather than its frequency in the parasite population. Due to multiple infections, the absence and presence of haplotypes in infections is in general ambiguous. Hence, prevalence is an unobservable quantity (unobservable prevalence). However, estimates for (unobservable) prevalence are readily derived as plug-in estimates from the MLE and (16c). In fact, these estimates are very accurate (Figures 10–13), except if haplotypes are rare and sample size is small, in which case the prevalence is underestimated (cf. Figures 12B, 13B).

In practice, prevalence is often estimated as the conditional prevalence (21). This quantity substantially underestimates the (unobservable) prevalence (Figures 10–13). Hence, it should not be used as a proxy for prevalence. However, although the conditional prevalence is not recommendable, it can be accurately recaptured from the MLE and (21).

In the absence of a statistical model, haplotype frequencies can be estimated by normalizing the conditional prevalences of the haplotypes (relative unambiguous prevalence) (22b). These are undesirable *ad-hoc* estimates, because they are biased (Figures 10–13). Particularly unfortunate is that bias depends on MOI. The larger the mean MOI, the larger the bias. However, it is rather unpredictable whether a particular haplotype's frequency is over- or underestimated (Figures 10–13). This depends on the true haplotype frequencies and MOI. The relative bias can be accurately obtained as a plug-in estimate from the MLE and (22b).

## 4. Discussion

Public health strategies are increasingly relying on genomic/molecular surveillance to monitor infectious diseases [cf. (2, 3)]. This is particularly true for malaria, where molecular surveillance is a standard approach to monitor pathogen variants, which are associated with drug resistance, or jeopardize reliable diagnostics (e.g., *P. falciparum* variants with deletions in the HRP2/3 genes, which can lead to false-negative rapid diagnostic test results [cf. (52)]. Moreover, patterns of transmission, disease exposure, or the evolutionary genetics can be ascertained by studying genomic/molecular data [cf. (53–55)].

A usual problem in molecular surveillance in the context of malaria is the presence of several genetically distinct parasite haplotypes within an infection—this is particularly common in areas of high transmission [cf. (41, 54)]. Unfortunately, in such cases, usual molecular methods provide only ambiguous information concerning the haplotypes present in infections [cf. (15)]. Namely, molecular information is typically unphased. In the case of, e.g., antimalarial drug resistance, precise estimates of the frequency of particular haplotypes and the likelihood that they are observed in an infection (prevalence) are required. This requires sophisticated statistical models that resolve the underlying ambiguity in the observations.

Here, we introduced a statistical model to estimate the frequencies and prevalence of pathogen haplotypes from molecular data. More precisely, hosts can be super-infected several times with the same or different pathogenic variants. The number of super-infections is referred to as multiplicity of infection (MOI). Concerning the genetic architecture of pathogen variants, we assumed the pathogen to be haploid, and haplotypes to be determined by *n* biallelic loci. Typical applications are malaria parasites associated with drug resistance, where loci correspond to specific codons in one or more genes, e.g., codons 51, 59, 108, 164 in the *dhfr* locus and codons 436, 437, 540, 581, 613 in the *dhps* locus of *P. falciparum* associated with resistance to sulfadoxine-pyrimethamine (SP). The method is intended to derive frequencies of haplotypes which are “aggregates,” e.g., certain drug resistant haplotypes. It is not intended to characterize parasites at the individual level. Hence, the number of loci (*n*) should not be so large, that each haplotype occurs only once in the overall sample. In our simulations, we used up to *n* = 10. However, the method is not limited to 10 loci. Importantly, *n* is the number of loci, which are found polymporphic in the data, as monomorphic loci can be dropped.

We suggested a maximum-likelihood estimation of haplotype frequencies and the distribution of MOI, assuming an underlying conditional Poisson distribution. As in (28), we employ the expectation-maximization (EM) algorithm to derive the maximum-likelihood estimate (MLE). However, Li et al. (28) provided only a general form, which can be derived numerically by brute force. We derived a more explicit version that allows an efficient implementation, which is provided as an easy-to-use R script. Importantly, based on the statistical framework, we provided explicit expressions for prevalence.

Based on this, the MLE can be used as a plug-in statistic, to provide estimates for prevalence. For instance, in the context of antimalarial drug resistance, the prevalence of drug-resistant haplotypes is a more relevant quantity regarding disease outcomes. The frequency of a haplotype is its relative abundance in the pathogen population, whereas prevalence is the probability that the haplotype occurs in an infection. Estimating prevalence is notoriously difficult. If transmission is low, i.e., the average MOI is small, prevalence and frequency almost coincide. However, if transmission is high (large average MOI), prevalence can be substantially higher than frequency. If MOI is high, several pathogen haplotypes commonly occur in infections. However, if molecular methods provide only unphased information, they do not allow to directly observe haplotypes in such infections [cf. (16)]. Therefore, a statistical model is required to resolve this ambiguity. *Ad-hoc* methods to estimate prevalence do not require an explicit statistical model. However, they must necessarily be based only on unambiguous information. We investigated the deviations of conditional prevalence (conditioned on unambiguous information) and prevalence and concluded that *ad-hoc* approximations to prevalence might substantially underestimate the true prevalence. Indeed it might be highly problematic for treatment policies if prevalence of drug-resistant pathogen variants are underestimated.

Here, we applied the method to an empirical dataset of mutations associated with SP resistance from Cameroon in the years 2001/2002 and 2004/2005. Furthermore, we investigated the performance of the MLE in terms of bias and variance. In general, the estimates of the haplotype frequencies have little bias. Bias tends to be higher for higher average MOI. Moreover, in relative terms, bias is higher for rare haplotypes. The MOI parameter has a higher bias than the frequency estimates, particularly if average MOI is high. However, bias decreases quickly with sample size. Also, the variance of the estimator, in terms of the coefficient of variation tends to be small. Due to the good performance of the estimator, also the estimates of prevalence are reliable.

Although the method performs well, it has certain limitations. So far it (i) is restricted to SNP data (or biallelic data); (ii) does not account for missing information; (iii) does not incorporate errors in the data (due to the molecular methods used to generate the data); (iv) does not take relatedness between pathogen variants and co-transmissions into account. The first three limitations are however justified by the curse of dimensionality. Assuming *n* loci with three instead of two alleles would result in 3^*n*^ rather than 2^*n*^ possible haplotypes. For *n* = 5 loci this amounts to 243 rather than 32 haplotypes. Importantly, the biallelic genetic architecture is justified by the popularity of SNP data. In the case of malaria, sample sizes of *N* = 50 to *N* = 500 are realistic. With *n* = 10 loci, 1,024 haplotypes are possible. Hence, the number of parameters would by far exceed the sample size. Importantly, in practice, not all of the 1,024 possible haplotypes are compatible with the data. Hence, only a subset of haplotypes is relevant, rendering a realistic sample size to be adequate. However, when aiming to incorporate missing information and errors, all possible haplotypes have to be considered in a statistical model, with the majority of them being irrelevant. An exact statistical model will be hopelessly over-parameterized if the number of considered loci is large. Therefore, approximate models would need to be considered, which disregard infrequent haplotypes. The fourth limitation deserves particular attention. With genomic data becoming more available in molecular surveillance, methods have been developed to account for relatedness of pathogen variants within infections [cf. (35–40)]. Genetic relatedness is informative on transmission dynamics, i.e., knowledge of whether pathogen variants co-occurring in a single infection are identical by state or identical by descent, or whether they were co-transmitted together rather than sequentially is informative on possible routes of transmission (35, 40). Such methods however require genomic information or at least larger SNP barcodes. Although genomic sequencing is becoming more affordable, obtaining such data requires efforts and resources, which are still not feasible in many settings. Note that in vector-borne diseases like malaria, genetic relatedness is only partially informative on transmission dynamics. Namely, it is unclear whether relatedness is caused by some mosquitoes infecting many hosts, or whether mosquitoes get infected by many hosts. Particularly, the admixture of the vector and host populations influence relatedness of the pathogen within the hosts and vectors. To an extreme, if the disease is transmitted mainly within households, i.e., the vectors are not well mixed with the host population, high relatedness of pathogen variants within infections is expected, independently of the overall transmission intensity. On the other hand, if infections within households are uncommon, less relatedness is expected. Hence, relatedness might be more informative on the routes of transmission than on transmission intensities themselves. In the latter case, the proposed approach to estimate MOI seems preferable. Some methods also estimate the relative abundance of haplotypes in an infection (40). Such information is important if there is evidence that the pathogenesis of the disease depends on the interactions of pathogen variants within the infection—especially, if the emphasis is on the clinical manifestation of the disease rather than on the pathogen population level. Notably, all methods that consider relatedness have their limitations. Since (due to the curse of dimensionality) they are not haplotype based, typically independence of genetic markers is assumed. For applications such as drug resistance in malaria, this assumption is not justified, such that a haplotype based approach as proposed here seems more appropriate. In vector-borne diseases, one of the main advantage of the proposed method, is that it does not require an explicit model of vector-host dynamics. Incorporating relatedness, tailored to the characteristics of the disease, would require imposing such model to be accurate. This, however, would require several assumptions, which might yet be poorly justified by empirical evidence. In case co-transmission of pathogen variants are important, it would be interesting to ascertain how well the proposed method performs. However, such assessment is notoriously difficult, because a true model for transmission needs to be specified based on empirical evidence.

In conclusion, we provided a method to estimate haplotype frequencies and prevalences alongside the distribution of MOI from malaria genetic data. The estimator shows convenient statistical properties and can be efficiently implemented. The estimator is implemented in an easy-to-use R script available on Github at https://github.com/Maths-against-Malaria/MultiLociBiallelicModel.git.

## Data availability statement

The original contributions presented in the study are included in the article/Supplementary material, further inquiries can be directed to the corresponding author/s.

## Author contributions

HT contributed in the study design, carried out the mathematical analysis, numerical implementation, data analysis, performed numerical simulations, created the graphics, and wrote the manuscript. KS contributed in designing the study, the mathematical analysis, supervised the numerical implementation, helped to design the numerical simulations, and participated in writing and correcting the manuscript. Both authors contributed to the article and approved the submitted version.

## Funding

This work was supported by grants of the German Academic Exchange (DAAD; https://www.daad.de/de/; Project-ID 57417782, Project-ID: 57599539), the Sächsisches Staatsministerium für Wissenschaft, Kultur und Tourismus and Sächsische Aufbaubank – Förderbank (SMWK-SAB; https://www.smwk.sachsen.de/; https://www.sab.sachsen.de/; project Innovationsvorhaben zur Profilschärfung an Hochschulen für angewandte Wissenschaften, Project-ID 100257255; project Innovationsvorhaben zur Profilschärfung 2022, Project-ID: 100613388), the Federal Ministry of Education and Research (BMBF) and the DLR (Project-ID 01DQ20002; https://www.bmbf.de/; https://www.dlr.de/). The funders had no role in study design, data collection and analysis, decision to publish, or preparation of the manuscript.

## Conflict of interest

The authors declare that the research was conducted in the absence of any commercial or financial relationships that could be construed as a potential conflict of interest.

## Publisher's note

All claims expressed in this article are solely those of the authors and do not necessarily represent those of their affiliated organizations, or those of the publisher, the editors and the reviewers. Any product that may be evaluated in this article, or claim that may be made by its manufacturer, is not guaranteed or endorsed by the publisher.
