# Supplementary figures and images for: A maximum-likelihood method to estimate haplotype frequencies and prevalence alongside multiplicity of infection from SNP data
Source: Front Epidemiol. 2022 Sep 23;2:943625. doi: 10.3389/fepid.2022.943625 (PMC10911023; doi:10.3389/fepid.2022.943625)

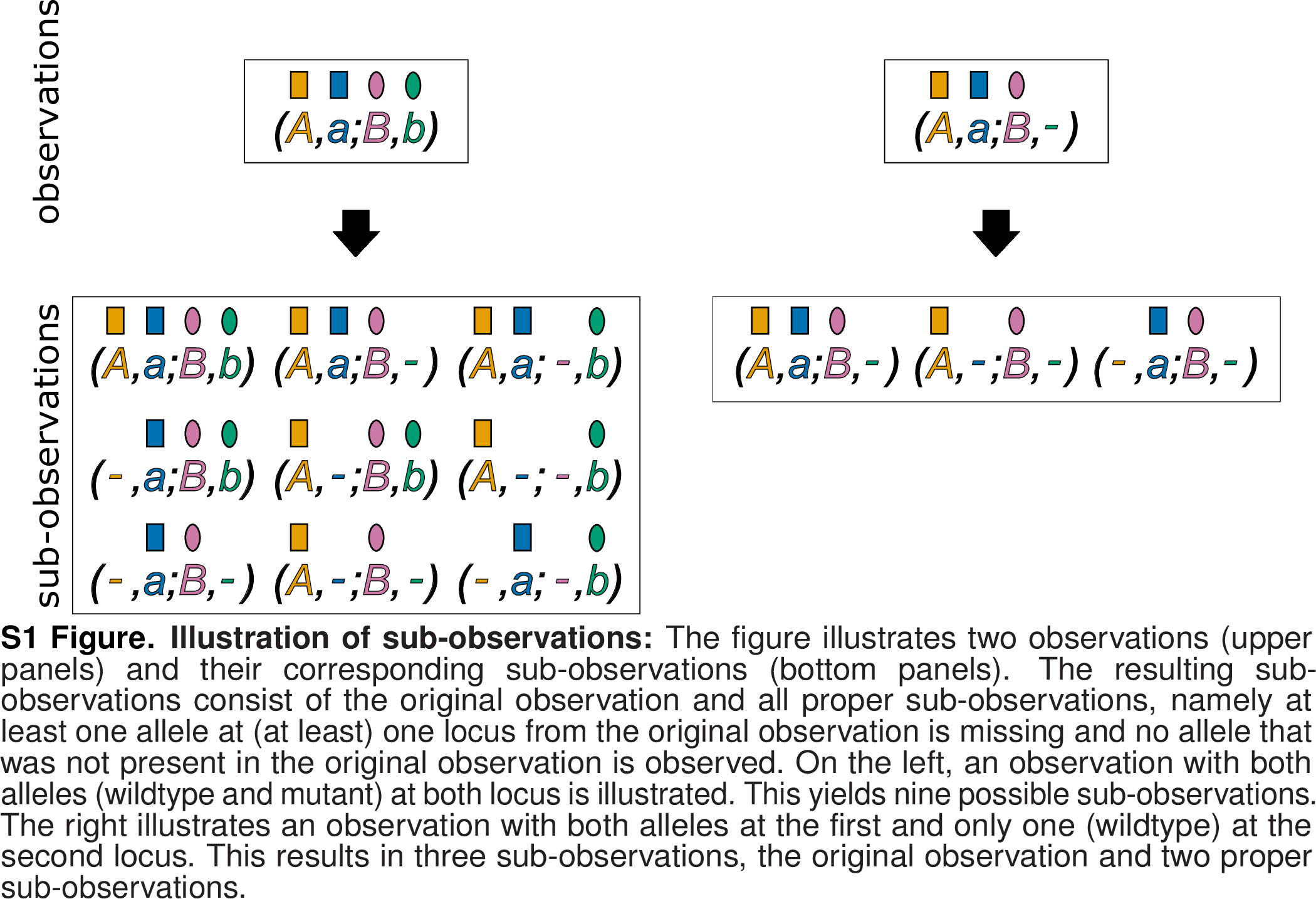

Supplement: Supplementary file 1 [file Image_1.tif]
