# Supplementary material for: A maximum-likelihood method to estimate haplotype frequencies and prevalence alongside multiplicity of infection from SNP data
Source: Front Epidemiol. 2022 Sep 23;2:943625. doi: 10.3389/fepid.2022.943625 (PMC10911023; doi:10.3389/fepid.2022.943625)
Supplement: Supplementary file 5 [file Data_Sheet_2.pdf]

1 USER MANUAL

The R script ‘SNPModel.R’ contains the necessary functions to replicate the results of the main manuscript and to estimate haplotype frequencies, prevalence, and MOI from empirical data. The required data structure and use of the main functions are described below. The R script ‘SNP_MLE.R’ is a template script that contains the code described below.

The code and some dataset examples are available on Github at `https://github.com/Maths-against-Malaria/MultiLociBiallelicModel.git`.

1.1 Loading the R script

To load the R script, store it in a custom directory “<PATH>/SNPModel.R” and load it. If the script is stored in the path “/home/janedoe/Documents”, the following code will load the R script:

```
# Load external resources
    source("/home/janedoe/Documents/SNPModel.R")
```

1.2 Data structure

The method is applicable to biallelic data, e.g., SNP data. Data has to be entered in a standard format. Considering n biallelic markers (SNPs), an observation (sample) corresponds to a vector with n elements. The kth element indicates whether only the wildtype allele (=0), only the mutant allele (=1), or both wildype and mutant (=2) are observed at the kth biallelic maker. A data set of sample size N is represented by an $N \times n$ matrix with entries 0, 1, or 2. The entry in the lth row and kth column indicates which alleles are observed in sample l at marker k.

A dataset containing $N = 100$ samples characterized by $n = 5$ loci has the following form:

ID	Marker1	Marker2	Marker3	Marker4	Marker5
ID1	2	2	0	2	2
ID2	2	0	1	0	2
ID3	2	2	2	0	0
⋮	⋮	⋮	⋮	⋮	⋮
ID99	2	2	0	0	0
ID100	0	0	0	0	0

The column containing the row labels (sample IDs) and the row containing the column labels (marker IDs) are optional.

Data can be entered directly in R as an array with N rows and n columns or imported from typical formats, e.g., ’.xlsx’, ’.xls’,’.csv’, ’.txt’.

1.3 Importing data

Assume the data is available as an ’.xlsx’ file. An example data set is provided in the supplement (‘exampleData.xlsx’).

Assume the file ‘exampleData.xlsx’ is stored in the directory “/home/janedoe/Documents/”. The file can be imported with the R package ‘xlsx’. If the package is not yet installed run the code:

```
# Install the necessary packages
    install.packages('xlsx')
```

After successfully installing the package ‘xlsx’ data can be imported using the following code:

```
# Load libraries
    library(xlsx)

# Import the data
    DATA <- read.xlsx("/home/janedoe/Documents/example.xlsx",1,header=TRUE)
```

This code loads the dataset “/home/janedoe/Documents/example.xlsx” into the array ‘DATA’. The path “/home/janedoe/Documents/example.xlsx” has to be adjusted properly by the user (general syntax: “<PATH>/<FILENAME.xlsx>”). If the first line in the ‘.xlsx’ file does not contain the column names, the option ‘header’ has to be set to ‘header=FALSE’.

Note that different R packages are capable of importing ‘.xlsx’ files. The package ‘xlsx’ is based on ‘rJava’, which requires a Java installation. In case of problems, it is recommendable to import the data from a ‘.txt’ file or ‘.csv’ file using the functions ‘read.csv’ or ‘read.table’, respectively.

2 ESTIMATING FREQUENCIES, PREVALENCE, AND MOI

The function ‘mle(<DATA>,. . .)’ will derive the maximum likelihood estimate (MLE). The function outputs a 3-element list. The first element is the MLE for the MOI parameter λ, the second element is the matrix that contains the MLE for the haplotype frequencies. Only the frequencies larger than 0 are returned. The column labels indicate the corresponding haplotypes (0=wildtype allele, 1=mutant allele). The third element is a matrix that contains the haplotypes with strictly positive frequency estimates.

Assume the data is given by the array ‘DATA’. Further assume that the first column of ‘DATA’ contains sample IDs. The following code will output the maximum likelihood estimate of the data ‘DATA’:

```
# Obtain MLEs
    mle(DATA, id=TRUE, plugin=NULL)
```

When ‘DATA’ is the data provided by ‘example.xlsx’, it will result in the following output:

```
> mle(DATA, id=TRUE, plugin=NULL)
$lambda
0.5537991

$p
             00        01        10        11
[1,] 0.221456 0.3056585 0.2158763 0.2570091

$haplotypes
     [,1] [,2]
[1,]    0    0
[2,]    0    1
```

```
[3,]    1    0
[4,]    1    1
```

If the first column of the dataset does not contain the sample IDs, the option 'id=FALSE' has to be specified. If one does not want to estimate the Poisson parameter λ but use a plug-in estimate instead, the option 'plugin = λ' needs to be specified. For instance if one wishes to use $\lambda = 0.7$ as plug in estimate for the Poisson parameter, the following code needs to be run:

```
> mle(DATA, id=TRUE, plugin=0.7)
$lambda
0.7

$p
           00         01         10         11
[1,] 0.220933  0.3067905  0.2151696  0.2571069

$haplotypes
     [,1] [,2]
[1,]    0    0
[2,]    0    1
[3,]    1    0
[4,]    1    1
```

Biased corrected estimates can be obtained by setting the option 'BC = TRUE'. The default is a bootstrap bias correction (method="bootstrap") based on $= 10\,000$ boostrap replicates (Bbias $= 10\,000$). Alternatively a jackknife bias correction can be obtained by setting the option method="jackknife". To obtain the bias corrected estimates using the bootstrap method, one should run the following code:

```
> mle(Data, id=TRUE, plugin=NULL, BC=TRUE, method='bootstrap')
$lambda
0.5483354

$p
           00         01         10         11
[1,] 0.221104  0.3060268  0.2160371  0.2568322

$haplotypes
     [,1] [,2]
[1,]    0    0
[2,]    0    1
[3,]    1    0
[4,]    1    1
```

Moreover, bootstrap confidence interval are outputted alongside the estimates if the option 'CI = TRUE' is specified. The default are $B = 10\,000$ bootstrap repeats. To obtain the estimates of MOI and haplotype frequencies with their corresponding 95% confidence interval, one should run the following code:

```
> mle(DATA, id=TRUE, plugin=NULL, CI=TRUE)

$lambda
                2.5%     97.5%
0.5537991 0.3490226 0.7985430

$p
                   2.5%     97.5%
00 0.2214560 0.1452902 0.3023012
01 0.3056585 0.2237450 0.3898143
10 0.2158763 0.1412364 0.2954705
11 0.2570091 0.1811485 0.3368962

$haplotypes
     [,1] [,2]
[1,]    0    0
[2,]    0    1
[3,]    1    0
[4,]    1    1
```

To change the number of bootstrap repeats to X the option $B = X$ has to be specified. To obtain $(1-\alpha)$-level confidence intervals, the option $alpha = \alpha$ needs to be specified. The following code estimates of MOI and haplotype frequencies with 90% bootstrap confidence intervals based on 15000 bootstrap repeats:

```
> mle(DATA, id=TRUE, plugin=NULL, CI=TRUE, B=15000, alpha=0.1)

$lambda
                  5%       95%
0.5537991 0.3781955 0.7562267

$p
                     5%       95%
00 0.2214560 0.1571200 0.2892539
01 0.3056585 0.2355181 0.3762861
10 0.2158763 0.1530241 0.2823532
11 0.2570091 0.1934159 0.3228874

$haplotypes
     [,1] [,2]
[1,]    0    0
[2,]    0    1
[3,]    1    0
[4,]    1    1
```

The MLEs outputted by the function ‘mle(<DATA>,. . .)’ can be used as input for the functions ‘estunobsprev(<MLE>)’ and ‘estcondprev(<MLE>)’ to estimate unobserved and conditional prevalence, respectively. Importantly, only the estimates without confidence intervals are used to estimate prevalence. This can be done with the following code:

```
# Find the MLEs
    est <- mle(DATA, id=TRUE, plugin=NULL, CI=FALSE)
# Estimate prevalence
    ## Unobservable prevalence
    unobsprev <- estunobsprev(est)

    ## Conditional prevalence
    condprev <- estcondprev(est)
```

If ‘est’ is the output of ‘mle(<DATA>,. . .)’ assuming that ‘DATA’ is the data provided by the file ‘example.xlsx’, the estimates of the unobserved and conditional prevalence are:

```
> estunobsprev(est)
            00        01        10        11
[1,] 0.2714241 0.3661996 0.2649863 0.3119812

> estcondprev(est)
            00        01        10        11
[1,] 0.2504816 0.3465174 0.2385816 0.2936344
```

If a plug-in estimate was used for λ in the function ‘mle’, and the output is used to calculate the unobserved or conditional prevalence, the plug in estimate will be used automatically.

Relative prevalence is estimated from ‘DATA’ by the function ‘relprev(<DATA>,. . .)’. The code to obtain the estimates of prevalence is:

```
    ## Relative prevalence
    relprev <- estrelprev(DATA, id=TRUE)
```

If ‘DATA’ is provided by ‘example.xlsx’, the estimates for relative are:

```
> estrelprev(DATA, id=TRUE)
            00       10        01        11
[1,] 0.2222222 0.212963 0.3055556 0.2592593
```

If the first column of the dataset does not contain the sample IDs, the option ‘id=FALSE’ has to be specified.

2.1 MOI estimates per sample

Based on estimates $\hat{\lambda}$, and $\hat{\boldsymbol{p}}$ for the MOI parameter λ and haplotype frequencies $\boldsymbol{p}$, MOI of a given sample $\boldsymbol{x}$ can be estimated using the function ‘samplwiseMOI(M=10, X, est)’. The inputs of the function are: (i) the sample $\boldsymbol{x}$ for which the underlying MOI should be estimated, (iii) the parameter estimates as outputted from the function ‘mle(<DATA>,...)’, and (iii) the maximum value of MOI M (default $M = 10$). The

output is a list containing the estimated MOI for the sample and an $M \times 2$ matrix in which the first column contains values of $m = 1, \ldots, M$, and the second column the corresponding probabilities $P(m|\boldsymbol{x}, \hat{\lambda}, \hat{\boldsymbol{p}})$. The estimated samplewise MOI is the value of m, which maximizes the probability $P(m|\boldsymbol{x}, \hat{\lambda}, \hat{\boldsymbol{p}})$.

Assuming the sample $\boldsymbol{x} = (2, 0)$ sampled from the same population as the samples in DATA, to obtain an estimate of MOI underlying $\boldsymbol{x}$, one should run the following code:

```
> X <- c(2,0)
> samplwiseMOI(X, est, M=10)
$MOI
2

$probability
      m            p
 [1,]  1 0.000000e+00
 [2,]  2 8.848659e-01
 [3,]  3 1.071547e-01
 [4,]  4 7.569584e-03
 [5,]  5 3.928643e-04
 [6,]  6 1.638734e-05
 [7,]  7 5.761477e-07
 [8,]  8 1.758121e-08
 [9,]  9 4.749876e-10
[10,] 10 1.152658e-11
```

3 GENERATING SIMULATED DATA

Simulated data can be generated using the function “datasetgen(P,lambda,N,n)”. The required arguments are the haplotype frequency distribution $\boldsymbol{p}$ of length 2^n, the MOI parameter λ, the sample size N, and the number n of bialellic loci, which determine the haplotypes. For example, when considering $n = 2$ loci, haplotype frequency distribution $\boldsymbol{p} = (0.45, 0.25, 0.2, 0.1)$, MOI parameter $\lambda = 0.5$ a dataset of sample size $N = 100$ is generated with the following code:

```
# Generate dataset
    datasetgen(c(0.45,0.25,0.20,0.10), 0.5, 100, 2)
```

The output is a random $N \times n$ matrix with entries corresponding to the data structure described above.

Regarding the frequency vector $\boldsymbol{p}$, the haplotypes have to be in a specific order. The required order is obtained by the function “hapl(n)”. The function outputs a $2^n \times n$ matrix with entries 0 and 1, where each row is the representation of a haplotype (0=wildtype allele, 1=mutant allele). Each row can be interpreted as the binary representation (plus one) of the index of the haplotype. For example for $n = 2$ loci the code outputs the representation of all 4 possible haplotypes:

```
> hapl(2)
     [,1] [,2]
[1,]    0    0
[2,]    0    1
```

```
[3 ,]    1    0
[4 ,]    1    1
```

This indicates that haplotype (0,0) is represented by 1, haplotype (0,1) by 2, haplotype (1,0) by 3, and haplotype (1,1) by 4. Hence in the above code example $\boldsymbol{p} = (0.45, 0.25, 0.2, 0.1)$ indicates that haplotype (0,0) has frequency $p_1 = 0.45$, haplotype (0,1) has frequency $p_2 = 0.25$ haplotype (1,0) has frequency $p_3 = 0.2$ haplotype (1,1) has frequency $p_1 = 0.1$.
