# Supplementary material for: A maximum-likelihood method to estimate haplotype frequencies and prevalence alongside multiplicity of infection from SNP data
Source: Front Epidemiol. 2022 Sep 23;2:943625. doi: 10.3389/fepid.2022.943625 (PMC10911023; doi:10.3389/fepid.2022.943625)
Supplement: Supplementary file 7 [file Data_Sheet_4.pdf]

1 MATHEMATICAL APPENDIX

1.1 Prevalence estimates

First, we derive expression (19b) for the probability to observe haplotype $\boldsymbol{h}$ in an unambiguous observation, $r_{\boldsymbol{h}}$. Recall the definition of the set $U_{\boldsymbol{h}}$ of all haplotype $\boldsymbol{i}$, which yield unambiguous observations together with $\boldsymbol{h}$, defined in (18). Note that there are exactly n haplotypes $\boldsymbol{i}$ such that $\boldsymbol{i} \in U_{\boldsymbol{h}}$. Formally, we have Some rearrangement of (19a) in order to use the binomial theorem gives

$$\begin{aligned} r_{\boldsymbol{h}} &= \sum_{m=1}^{\infty} \kappa_m \sum_{\boldsymbol{i} \in U_{\boldsymbol{h}}} \sum_{m_{\boldsymbol{i}}=1}^{m-1} \binom{m}{m_{\boldsymbol{i}}} p_{\boldsymbol{i}}^{m_{\boldsymbol{i}}} p_{\boldsymbol{h}}^{m-m_{\boldsymbol{i}}} + \sum_{m=1}^{\infty} \kappa_m p_{\boldsymbol{h}}^m \\ &= \sum_{m=1}^{\infty} \kappa_m \sum_{\boldsymbol{i} \in U_{\boldsymbol{h}}} \sum_{m_{\boldsymbol{i}}=0}^{m} \binom{m}{m_{\boldsymbol{i}}} p_{\boldsymbol{i}}^{m_{\boldsymbol{i}}} p_{\boldsymbol{h}}^{m-m_{\boldsymbol{i}}} - \sum_{m=1}^{\infty} \kappa_m \sum_{\boldsymbol{i} \in U_{\boldsymbol{h}}} p_{\boldsymbol{i}}^m - (n-1) \sum_{m=1}^{\infty} \kappa_m p_{\boldsymbol{h}}^m \\ &= \sum_{\boldsymbol{i} \in U_{\boldsymbol{h}}} \sum_{m=1}^{\infty} \kappa_m (p_{\boldsymbol{h}} + p_{\boldsymbol{i}})^m - \sum_{\boldsymbol{i} \in U_{\boldsymbol{h}}} \sum_{m=1}^{\infty} \kappa_m p_{\boldsymbol{i}}^m - (n-1) \sum_{m=1}^{\infty} \kappa_m p_{\boldsymbol{h}}^m. \end{aligned}$$

Using the definition of the PGF (1b) yields

$$r_{\boldsymbol{h}} = \sum_{\boldsymbol{i} \in U_{\boldsymbol{h}}} \Big[G(p_{\boldsymbol{h}} + p_{\boldsymbol{i}}) - G(p_{\boldsymbol{i}}) \Big] - (n-1) G(p_{\boldsymbol{h}}). \tag{A.1a}$$

Next, the probability of all unambiguous observations $P(\tilde{\mathscr{O}})$ is derived. Importantly, it is not simply obtained as the sum over all quantities $r_{\boldsymbol{h}}$. The reason is that the sets $U_{\boldsymbol{h}}$ are not pairwise disjoint. Namely, if for two haplotypes $\boldsymbol{h}$ and $\boldsymbol{i}$, $\boldsymbol{i} \in U_{\boldsymbol{h}}$, then $\boldsymbol{h} \in U_{\boldsymbol{i}}$. Note that each haplotype $\boldsymbol{h}$ is contained in exactly n sets $U_{\boldsymbol{i}}$. When using the sets $U_{\boldsymbol{h}}$ again to derive $P(\tilde{\mathscr{O}})$ it is important to discard all the repeating outcomes that would be counted multiple times (i.e., the double infections with $\boldsymbol{h}$ and $\boldsymbol{i} \in U_{\boldsymbol{h}}$ and the single infections with $\boldsymbol{h}$ have to be counted only once). Considering this, the probability $P(\tilde{\mathscr{O}})$ can be written as

$$\begin{aligned} P(\tilde{\mathscr{O}}) :&= \sum_{\boldsymbol{h} \in \mathscr{H}} \left[\frac{1}{2} \sum_{m=1}^{\infty} \kappa_m \sum_{\boldsymbol{i} \in U_{\boldsymbol{h}}} \sum_{k=0}^{m} \binom{m}{k} p_{\boldsymbol{h}}^k p_{\boldsymbol{i}}^{m-k} - \frac{1}{2} \sum_{m=1}^{\infty} \kappa_m \sum_{\boldsymbol{i} \in U_{\boldsymbol{h}}} p_{\boldsymbol{i}}^m - \Big(\frac{n}{2} - 1\Big) \sum_{m=1}^{\infty} \kappa_m p_{\boldsymbol{h}}^m \right] \\ &= \sum_{\boldsymbol{h} \in \mathscr{H}} \left[\frac{1}{2} \sum_{\boldsymbol{i} \in U_{\boldsymbol{h}}} \sum_{m=1}^{\infty} \kappa_m (p_{\boldsymbol{h}} + p_{\boldsymbol{i}})^m - \frac{1}{2} \sum_{\boldsymbol{i} \in U_{\boldsymbol{h}}} \sum_{m=1}^{\infty} \kappa_m p_{\boldsymbol{i}}^m - \big(\frac{n}{2} - 1\big) \sum_{m=1}^{\infty} \kappa_m p_{\boldsymbol{h}}^m \right]. \end{aligned}$$

Using the PGF again, one arrives at

$$P(\tilde{\mathscr{O}}) = \sum_{\boldsymbol{h} \in \mathscr{H}} \left[\frac{1}{2} \sum_{\boldsymbol{i} \in U_{\boldsymbol{h}}} \Big[G(p_{\boldsymbol{h}} + p_{\boldsymbol{i}}) - G(p_{\boldsymbol{i}}) \Big] - \Big(\frac{n}{2} - 1\Big) G(p_{\boldsymbol{h}}) \right].$$

1.2 Deriving the EM-algorithm

The EM-algorithm consists of two steps. First, we derive the E-step, followed by the M-step.

Expectation (E)-step

Each sample $\boldsymbol{x}^{(j)}$ in the dataset $\mathscr{X} = (\boldsymbol{x}^{(1)}, \ldots, \boldsymbol{x}^{(N)})$ is derived from a particular MOI vector $\boldsymbol{m}^{(j)}$ that is unobservable. We denote by $\mathscr{M} = (\boldsymbol{m}^{(1)}, \ldots, \boldsymbol{m}^{(N)})$ the MOI vectors associated with $\mathscr{X}$. Hence, the likelihood function $\ell_{\mathscr{X},\mathscr{M}}$ is defined as

$$\ell_{\mathscr{X},\mathscr{M}}(\boldsymbol{\theta}) = \prod_{j=1}^{N} P\big(\boldsymbol{x}^{(j)}, \boldsymbol{m}^{(j)} \big| \boldsymbol{\theta}\big). \tag{A.2}$$

The Q-function in step t is the expectation of the log-likelihood function of $\boldsymbol{\theta}$, given the observed and unobserved data $\mathscr{X}$ and $\mathscr{M}$ with respect to the conditional distribution of the unobserved data $\mathscr{M}$, given the observed data $\mathscr{X}$ and the parameter choice in the t-th step $\boldsymbol{\theta}_t$, i.e.,

$$\begin{aligned} Q(\boldsymbol{\theta} \,|\, \boldsymbol{\theta}_t) &= \mathbb{E}_{\mathscr{M}|\mathscr{X},\boldsymbol{\theta}_t}[\ell_{\mathscr{X},\mathscr{M}}(\boldsymbol{\theta})] \\ &= \mathbb{E}_{\mathscr{M}|\mathscr{X},\boldsymbol{\theta}_t}\Big[\log \prod_{j=1}^{N} P\big(\boldsymbol{x}^{(j)}, \boldsymbol{m}^{(j)} \big| \boldsymbol{\theta}\big)\Big] \\ &= \mathbb{E}_{\mathscr{M}|\mathscr{X},\boldsymbol{\theta}_t}\Big[\sum_{j=1}^{N} \log P\big(\boldsymbol{x}^{(j)}, \boldsymbol{m}^{(j)} \big| \boldsymbol{\theta}\big)\Big] \\ &= \sum_{j=1}^{N} \mathbb{E}_{\mathscr{M}|\mathscr{X},\boldsymbol{\theta}_t}\big[\log P\big(\boldsymbol{x}^{(j)}, \boldsymbol{m}^{(j)} \big| \boldsymbol{\theta}\big)\big]. \end{aligned}$$

Note that

$$\begin{aligned} \mathbb{E}_{\mathscr{M}|\mathscr{X},\boldsymbol{\theta}_t} \log P\big(\boldsymbol{x}^{(j)}, \boldsymbol{m}^{(j)} \big| \boldsymbol{\theta}\big) &= \sum_{\boldsymbol{m}^{(1)},\ldots,\boldsymbol{m}^{(N)}} \log P\big(\boldsymbol{x}^{(j)}, \boldsymbol{m}^{(j)} \big| \boldsymbol{\theta}\big) P\big(\boldsymbol{m}^{(1)}, \ldots, \boldsymbol{m}^{(N)} \big| \boldsymbol{x}^{(1)}, \ldots, \boldsymbol{x}^{(N)}, \boldsymbol{\theta}_t\big) \\ &= \sum_{\boldsymbol{m}^{(1)},\ldots,\boldsymbol{m}^{(N)}} \log P\big(\boldsymbol{x}^{(j)}, \boldsymbol{m}^{(j)} \big| \boldsymbol{\theta}\big) \frac{P\Big(\big(x^{(1)}, m^{(1)}\big), \ldots, \big(x^{(N)}, m^{(N)}\big) \big| \boldsymbol{\theta}_t\Big)}{P\big(\boldsymbol{x}^{(1)}, \ldots, \boldsymbol{x}^{(N)} \big| \boldsymbol{\theta}_t\big)}. \end{aligned}$$

The samples in the observed and unobserved datasets $\mathscr{X}$ and $\mathscr{M}$ are independent. Hence,

$$\mathbb{E}_{\mathscr{M}|\mathscr{X},\boldsymbol{\theta}_t} \log P\big(\boldsymbol{x}^{(j)}, \boldsymbol{m}^{(j)} \big| \boldsymbol{\theta}\big) = \sum_{\boldsymbol{m}^{(1)},\ldots,\boldsymbol{m}^{(N)}} \log P\big(\boldsymbol{x}^{(j)}, \boldsymbol{m}^{(j)} \big| \boldsymbol{\theta}\big) \prod_{l=1}^{N} \frac{P\big(\boldsymbol{x}^{(l)}, \boldsymbol{m}^{(l)} \big| \boldsymbol{\theta}_t\big)}{P\big(\boldsymbol{x}^{(l)} \big| \boldsymbol{\theta}_t\big)}. \tag{A.3}$$

The fractions of probabilities in the above product are actually conditional probabilities, namely

$$\frac{P\big(\boldsymbol{x}^{(l)}, \boldsymbol{m}^{(l)} \big| \boldsymbol{\theta}_t\big)}{P\big(\boldsymbol{x}^{(l)} \big| \boldsymbol{\theta}_t\big)} = \frac{P\big(\boldsymbol{x}^{(l)}, \boldsymbol{m}^{(l)}, \boldsymbol{\theta}_t\big) P\big(\boldsymbol{\theta}_t\big)}{P\big(\boldsymbol{x}^{(l)}, \boldsymbol{\theta}_t\big) P\big(\boldsymbol{\theta}_t\big)} = \frac{P\big(\boldsymbol{x}^{(l)}, \boldsymbol{m}^{(l)}, \boldsymbol{\theta}_t\big)}{P\big(\boldsymbol{x}^{(l)}, \boldsymbol{\theta}_t\big)} = P\big(\boldsymbol{m}^{(l)} \big| \boldsymbol{x}^{(l)}, \boldsymbol{\theta}_t\big).$$

Consequently, (A.3) simplifies to

$$\mathbb{E}_{\mathscr{M}|\mathscr{X},\boldsymbol{\theta}_t} \log P\big(\boldsymbol{x}^{(j)}, \boldsymbol{m}^{(j)}|\boldsymbol{\theta}\big) = \sum_{\boldsymbol{m}^{(1)},\ldots,\boldsymbol{m}^{(N)}} \log P\big(\boldsymbol{x}^{(j)}, \boldsymbol{m}^{(j)}|\boldsymbol{\theta}\big) \prod_{l=1}^{N} P\big(\boldsymbol{m}^{(l)}|\boldsymbol{x}^{(l)}, \boldsymbol{\theta}_t\big). \tag{A.4}$$

Furthermore,

$$\mathbb{E}_{\boldsymbol{m}^{(j)}|\boldsymbol{x}^{(j)},\boldsymbol{\theta}_t} \log P\big(\boldsymbol{x}^{(j)}, \boldsymbol{m}^{(j)}\big) = \sum_{\boldsymbol{m}^{(j)}} \log P\big(\boldsymbol{x}^{(j)}, \boldsymbol{m}^{(j)}|\boldsymbol{\theta}\big) P\big(\boldsymbol{m}^{(j)}|\boldsymbol{x}^{(j)}, \boldsymbol{\theta}_t\big). \tag{A.5}$$

By rearranging the sum in (A.4) such that the innermost sum runs over all possible vectors $\boldsymbol{m}^{(j)}$ and using (A.5), one arrives at

$$\begin{aligned}
\mathbb{E}_{\mathscr{M}|\mathscr{X},\boldsymbol{\theta}_t} \log P\big(\boldsymbol{x}^{(j)}, \boldsymbol{m}^{(j)}|\boldsymbol{\theta}\big) &= \sum_{\substack{\boldsymbol{m}^{(1)},\ldots,\boldsymbol{m}^{(j-1)},\\ \boldsymbol{m}^{(j+1)},\ldots,\boldsymbol{m}^{(N)}}} \mathbb{E}_{\boldsymbol{m}^{(j)}|\boldsymbol{x}^{(j)},\boldsymbol{\theta}_t}\Big[\log P\big(\boldsymbol{x}^{(j)}, \boldsymbol{m}^{(j)}|\boldsymbol{\theta}\big)\Big] \prod_{\substack{l=1\\ l\neq j}}^{N} P\big(\boldsymbol{m}^{(l)}|\boldsymbol{x}^{(l)}, \boldsymbol{\theta}_t\big) \\
&= \mathbb{E}_{\boldsymbol{m}^{(j)}|\boldsymbol{x}^{(j)},\boldsymbol{\theta}_t}\Big[\log P\big(\boldsymbol{x}^{(j)}, \boldsymbol{m}^{(j)}|\boldsymbol{\theta}\big)\Big] \sum_{\substack{\boldsymbol{m}^{(1)},\ldots,\boldsymbol{m}^{(j-1)},\\ \boldsymbol{m}^{(j+1)},\ldots,\boldsymbol{m}^{(N)}}} \prod_{\substack{l=1\\ l\neq j}}^{N} P\big(\boldsymbol{m}^{(l)}|\boldsymbol{x}^{(l)}, \boldsymbol{\theta}_t\big).
\end{aligned} \tag{A.6}$$

Since for all l

$$\sum_{\boldsymbol{m}^{(l)}} P\big(\boldsymbol{m}^{(l)}|\boldsymbol{x}^{(l)}, \boldsymbol{\theta}_t\big) = 1,$$

rearranging the sum in (A.6) gives

$$\sum_{\substack{\boldsymbol{m}^{(1)},\ldots,\boldsymbol{m}^{(j-1)},\\ \boldsymbol{m}^{(j+1)},\ldots,\boldsymbol{m}^{(N)}}} \prod_{\substack{l=1\\ l\neq j}}^{N} P\big(\boldsymbol{m}^{(l)}|\boldsymbol{x}^{(l)}, \boldsymbol{\theta}_t\big) = 1.$$

Therefore,

$$\mathbb{E}_{\mathscr{M}|\mathscr{X},\boldsymbol{\theta}_t} \log P\big(\boldsymbol{x}^{(j)}, \boldsymbol{m}^{(j)}|\boldsymbol{\theta}\big) = \mathbb{E}_{\boldsymbol{m}^{(j)}|\boldsymbol{x}^{(j)},\boldsymbol{\theta}_t} \log P\big(\boldsymbol{x}^{(j)}, \boldsymbol{m}^{(j)}|\boldsymbol{\theta}\big). \tag{A.7}$$

Hence, the Q-function becomes

$$Q(\boldsymbol{\theta}\,|\,\boldsymbol{\theta}_t) = \sum_{j=1}^{N} \mathbb{E}_{\boldsymbol{m}^{(j)}|\boldsymbol{x}^{(j)},\boldsymbol{\theta}_t} \log P\big(\boldsymbol{x}^{(j)}, \boldsymbol{m}^{(j)}|\boldsymbol{\theta}\big). \tag{A.8}$$

Note that the same observation $\boldsymbol{x}$ might occur several times in the data. Let $n_{\boldsymbol{x}}$, be the number of times observation $\boldsymbol{x}$ occurs in the data $\mathscr{X}$. With this notation, the Q-function can be rewritten as

$$Q(\boldsymbol{\theta}\,|\,\boldsymbol{\theta}_t) = \sum_{\boldsymbol{x}\in\mathscr{O}} n_{\boldsymbol{x}} \mathbb{E}_{\boldsymbol{m}|\boldsymbol{x},\boldsymbol{\theta}_t}\big[\log P(\boldsymbol{x},\boldsymbol{m}|\boldsymbol{\theta})\big]. \tag{A.9a}$$

By defining

$$Q_{\boldsymbol{x}}(\boldsymbol{\theta}\,|\,\boldsymbol{\theta}_t) := \mathbb{E}_{\boldsymbol{m}|\boldsymbol{x},\boldsymbol{\theta}_t}\big[\log P(\boldsymbol{x},\boldsymbol{m}|\boldsymbol{\theta})\big], \tag{A.9b}$$

the Q-function becomes

$$Q(\boldsymbol{\theta}\,|\,\boldsymbol{\theta}_t) = \sum_{\boldsymbol{x}\in\mathscr{O}} n_{\boldsymbol{x}} Q_{\boldsymbol{x}}(\boldsymbol{\theta}\,|\,\boldsymbol{\theta}_t). \tag{A.9c}$$

To further simplify (A.9c), we first simplify the expressions (A.9b). Recalling (6b) we have

$$Q_{\boldsymbol{x}}(\boldsymbol{\theta}\,|\,\boldsymbol{\theta}_t) = \mathbb{E}_{\boldsymbol{m}|\boldsymbol{x},\boldsymbol{\theta}_t}\big[\log P(\boldsymbol{x},\boldsymbol{m}|\boldsymbol{\theta})\big] = \sum_{\boldsymbol{m}\in M_{\boldsymbol{x}}^{(m)}} P(\boldsymbol{m}|\boldsymbol{x},\boldsymbol{\theta}_t)\log P(\boldsymbol{x},\boldsymbol{m}|\boldsymbol{\theta}), \tag{A.10a}$$

where the second equation is satisfied because $P(\boldsymbol{m}|\boldsymbol{x},\boldsymbol{\theta}_t) = 0$ if $\boldsymbol{m} \notin M_{\boldsymbol{x}}^{(m)}$ (defined in equation 3a). As shown above, $P(\boldsymbol{m}|\boldsymbol{x},\boldsymbol{\theta}_t) = \dfrac{P(\boldsymbol{x},\boldsymbol{m}|\boldsymbol{\theta}_t)}{P(\boldsymbol{x}|\boldsymbol{\theta}_t)}$. Combining this with (A.10a) yields

$$\begin{aligned} Q_{\boldsymbol{x}}(\boldsymbol{\theta}\,|\,\boldsymbol{\theta}_t) &= \sum_{\boldsymbol{m}\in M_{\boldsymbol{x}}^{(m)}} \frac{P(\boldsymbol{x},\boldsymbol{m}|\boldsymbol{\theta}_t)}{P(\boldsymbol{x}|\boldsymbol{\theta}_t)}\log P(\boldsymbol{x},\boldsymbol{m}|\boldsymbol{\theta}) \\ &= \frac{1}{P(\boldsymbol{x}|\boldsymbol{\theta}_t)} \sum_{\boldsymbol{m}\in M_{\boldsymbol{x}}^{(m)}} P(\boldsymbol{x},\boldsymbol{m}|\boldsymbol{\theta}_t)\log P(\boldsymbol{x},\boldsymbol{m}|\boldsymbol{\theta}). \end{aligned} \tag{A.10b}$$

Because $P(\boldsymbol{x},\boldsymbol{m}|\boldsymbol{\theta}_t) = P(\boldsymbol{x}|\boldsymbol{m},\boldsymbol{\theta}_t)P(\boldsymbol{m}|\boldsymbol{\theta}_t)$,

$$Q_{\boldsymbol{x}}(\boldsymbol{\theta}\,|\,\boldsymbol{\theta}_t) = \frac{1}{P(\boldsymbol{x}|\boldsymbol{\theta}_t)} \sum_{\boldsymbol{m}\in M_{\boldsymbol{x}}^{(m)}} P(\boldsymbol{x}|\boldsymbol{m},\boldsymbol{\theta}_t)P(\boldsymbol{m}|\boldsymbol{\theta}_t)\log\big[P(\boldsymbol{x}|\boldsymbol{m},\boldsymbol{\theta})P(\boldsymbol{m}|\boldsymbol{\theta})\big]. \tag{A.10c}$$

The sum in (A.10d) runs over all $\boldsymbol{m} \in M_{\boldsymbol{x}}^{(m)}$. For $\boldsymbol{m} \in M_{\boldsymbol{x}}^{(m)}$, clearly $P(\boldsymbol{x}|\boldsymbol{m},\boldsymbol{\theta}_t) = 1$ holds, such that

$$Q_{\boldsymbol{x}}(\boldsymbol{\theta}\,|\,\boldsymbol{\theta}_t) = \frac{1}{P(\boldsymbol{x}|\boldsymbol{\theta}_t)} \sum_{\boldsymbol{m}\in M_{\boldsymbol{x}}^{(m)}} P(\boldsymbol{m}|\boldsymbol{\theta}_t)\log P(\boldsymbol{m}|\boldsymbol{\theta}). \tag{A.10d}$$

Furthermore, the theorem of total probability yields

$$P(\boldsymbol{m}|\boldsymbol{\theta}_t) = \sum_{m=1}^{\infty} P(\boldsymbol{m}|m,\boldsymbol{\theta}_t)P(m|\boldsymbol{\theta}_t) \tag{A.10e}$$

and we define $\kappa_m^{(t)} := P(m|\boldsymbol{\theta}_t)$ (note that $\kappa_m = P(m|\boldsymbol{\theta})$). This and (2) allow to rewrite (A.10d) as

$$
\begin{aligned}
Q_{\boldsymbol{x}}(\boldsymbol{\theta}\,|\,\boldsymbol{\theta}_t) &= \frac{1}{P(\boldsymbol{x}|\boldsymbol{\theta}_t)} \sum_{m=1}^{\infty} \sum_{\boldsymbol{m}\in M_{\boldsymbol{x}}^{(m)}} P(\boldsymbol{m}|m,\boldsymbol{\theta}_t)P(m|\boldsymbol{\theta}_t) \log\left[P(\boldsymbol{m}|m,\boldsymbol{\theta})P(m|\boldsymbol{\theta})\right] \\
&= \frac{1}{P(\boldsymbol{x}|\boldsymbol{\theta}_t)} \sum_{m=1}^{\infty} \sum_{\boldsymbol{m}\in M_{\boldsymbol{x}}^{(m)}} \kappa_m^{(t)} \binom{m}{\boldsymbol{m}} \boldsymbol{p}_{\boldsymbol{h},t}^{\boldsymbol{m}} \log\left[\kappa_m \binom{m}{\boldsymbol{m}} \boldsymbol{p}^{\boldsymbol{m}}\right].
\end{aligned}
\tag{A.10f}
$$

The inclusion-exclusion principle from (7c) leads to

$$
Q_{\boldsymbol{x}}(\boldsymbol{\theta}\,|\,\boldsymbol{\theta}_t) = \frac{1}{P(\boldsymbol{x}|\boldsymbol{\theta}_t)} \sum_{m=1}^{\infty} \kappa_m^{(t)} \sum_{\boldsymbol{y}\in\mathscr{A}_{\boldsymbol{x}}} (-1)^{N_{\boldsymbol{x}}-N_{\boldsymbol{y}}} \sum_{\substack{\boldsymbol{m}:|\boldsymbol{m}|=m \\ m_{\boldsymbol{h}}=0 \text{ if } \boldsymbol{h}\notin A_{\boldsymbol{y}}}} \binom{m}{\boldsymbol{m}} \boldsymbol{p}_t^{\boldsymbol{m}} \log\left[\kappa_m \binom{m}{\boldsymbol{m}} \boldsymbol{p}^{\boldsymbol{m}}\right],
\tag{A.11a}
$$

where $N_{\boldsymbol{x}}$ and $N_{\boldsymbol{y}}$ are defined above (7b). Therefore, multiplication with $P(\boldsymbol{x}\,|\,\boldsymbol{\theta}_t)$ gives

$$
\begin{aligned}
&P(\boldsymbol{x}\,|\,\boldsymbol{\theta}_t) Q_{\boldsymbol{x}}(\boldsymbol{\theta}\,|\,\boldsymbol{\theta}_t) \\
&\quad= \sum_{\boldsymbol{y}\in\mathscr{A}_{\boldsymbol{x}}} (-1)^{N_{\boldsymbol{x}}-N_{\boldsymbol{y}}} \sum_{m=1}^{\infty} \kappa_m^{(t)} \sum_{\substack{\boldsymbol{m}:|\boldsymbol{m}|=m \\ m_{\boldsymbol{h}}=0 \text{ if } \boldsymbol{h}\notin A_{\boldsymbol{y}}}} \binom{m}{\boldsymbol{m}} \boldsymbol{p}_t^{\boldsymbol{m}} \log\left[\kappa_m \binom{m}{\boldsymbol{m}} \boldsymbol{p}^{\boldsymbol{m}}\right] \\
&\quad= \sum_{\boldsymbol{y}\in\mathscr{A}_{\boldsymbol{x}}} (-1)^{N_{\boldsymbol{x}}-N_{\boldsymbol{y}}} \sum_{m=1}^{\infty} \kappa_m^{(t)} \sum_{\substack{\boldsymbol{m}:|\boldsymbol{m}|=m \\ m_{\boldsymbol{h}}=0 \text{ if } \boldsymbol{h}\notin A_{\boldsymbol{y}}}} \binom{m}{\boldsymbol{m}} \boldsymbol{p}_t^{\boldsymbol{m}} \left[\log \kappa_m + \log \binom{m}{\boldsymbol{m}} + \log \boldsymbol{p}^{\boldsymbol{m}}\right] \\
&\quad= \sum_{\boldsymbol{y}\in\mathscr{A}_{\boldsymbol{x}}} (-1)^{N_{\boldsymbol{x}}-N_{\boldsymbol{y}}} \sum_{m=1}^{\infty} \kappa_m^{(t)} \sum_{\substack{\boldsymbol{m}:|\boldsymbol{m}|=m \\ m_{\boldsymbol{h}}=0 \text{ if } \boldsymbol{h}\notin A_{\boldsymbol{y}}}} \binom{m}{\boldsymbol{m}} \boldsymbol{p}_t^{\boldsymbol{m}} \left[\log\left(\frac{1}{e^{\lambda}-1}\frac{\lambda^m}{m!}\right) + \log \binom{m}{\boldsymbol{m}} + \log \boldsymbol{p}^{\boldsymbol{m}}\right] \\
&\quad= \sum_{\boldsymbol{y}\in\mathscr{A}_{\boldsymbol{x}}} (-1)^{N_{\boldsymbol{x}}-N_{\boldsymbol{y}}} \sum_{m=1}^{\infty} \kappa_m^{(t)} \sum_{\substack{\boldsymbol{m}:|\boldsymbol{m}|=m \\ m_{\boldsymbol{h}}=0 \text{ if } \boldsymbol{h}\notin A_{\boldsymbol{y}}}} \binom{m}{\boldsymbol{m}} \boldsymbol{p}_t^{\boldsymbol{m}} \left[-\log(e^{\lambda}-1) + m\log\lambda + \log \boldsymbol{p}^{\boldsymbol{m}}\right] + \tilde{A}_t,
\end{aligned}
$$

where

$$
\tilde{A}_t = \sum_{\boldsymbol{y}\in\mathscr{A}_{\boldsymbol{x}}} (-1)^{N_{\boldsymbol{x}}-N_{\boldsymbol{y}}} \sum_{m=1}^{\infty} \kappa_m^{(t)} \sum_{\substack{\boldsymbol{m}:|\boldsymbol{m}|=m \\ m_{\boldsymbol{h}}=0 \text{ if } \boldsymbol{h}\notin A_{\boldsymbol{y}}}} \binom{m}{\boldsymbol{m}} \boldsymbol{p}_t^{\boldsymbol{m}} \left[-\log(m!) + \log \binom{m}{\boldsymbol{m}}\right],
$$

is independent of $\boldsymbol{\theta}$. Combining this with (A.11a) yields

$$\begin{aligned} P(\boldsymbol{x}\,|\,\boldsymbol{\theta}_t)Q_{\boldsymbol{x}}(\boldsymbol{\theta}\,|\,\boldsymbol{\theta}_t) = &- \sum_{\boldsymbol{y}\in\mathscr{A}_{\boldsymbol{x}}}(-1)^{N_{\boldsymbol{x}}-N_{\boldsymbol{y}}}\sum_{m=1}^{\infty}\kappa_m^{(t)}\sum_{\substack{\boldsymbol{m}:|\boldsymbol{m}|=m\\ m_{\boldsymbol{h}}=0 \text{ if } \boldsymbol{h}\notin A_{\boldsymbol{y}}}}\binom{m}{\boldsymbol{m}}\boldsymbol{p}_t^{\boldsymbol{m}}\log(e^{\lambda}-1) \\ &+ \sum_{\boldsymbol{y}\in\mathscr{A}_{\boldsymbol{x}}}(-1)^{N_{\boldsymbol{x}}-N_{\boldsymbol{y}}}\sum_{m=1}^{\infty}m\kappa_m^{(t)}\sum_{\substack{\boldsymbol{m}:|\boldsymbol{m}|=m\\ m_{\boldsymbol{h}}=0 \text{ if } \boldsymbol{h}\notin A_{\boldsymbol{y}}}}\binom{m}{\boldsymbol{m}}\boldsymbol{p}_t^{\boldsymbol{m}}\log\lambda \\ &+ \sum_{\boldsymbol{y}\in\mathscr{A}_{\boldsymbol{x}}}(-1)^{N_{\boldsymbol{x}}-N_{\boldsymbol{y}}}\sum_{m=1}^{\infty}\kappa_m^{(t)}\sum_{\boldsymbol{h}\in\mathscr{H}}\log p_{\boldsymbol{h}}\sum_{\substack{\boldsymbol{m}:|\boldsymbol{m}|=m\\ m_{\boldsymbol{i}}=0 \text{ if } \boldsymbol{i}\notin A_{\boldsymbol{y}}}}m_{\boldsymbol{h}}\binom{m}{\boldsymbol{m}}\boldsymbol{p}_t^{\boldsymbol{m}} + \tilde{A}_t. \end{aligned} \tag{A.11b}$$

From (8c) and the binomial theorem we obtain

$$\begin{aligned} P(\boldsymbol{x}\,|\,\boldsymbol{\theta}_t)Q_{\boldsymbol{x}}(\boldsymbol{\theta}\,|\,\boldsymbol{\theta}_t) = &- P(\boldsymbol{x}\,|\,\boldsymbol{\theta}_t)\log(e^{\lambda}-1) \\ &+ \sum_{\boldsymbol{y}\in\mathscr{A}_{\boldsymbol{x}}}(-1)^{N_{\boldsymbol{x}}-N_{\boldsymbol{y}}}\sum_{m=1}^{\infty}m\kappa_m^{(t)}\Big(\sum_{\boldsymbol{i}\in A_{\boldsymbol{y}}}p_{\boldsymbol{i}}^{(t)}\Big)^{m}\log\lambda \\ &+ \sum_{\boldsymbol{y}\in\mathscr{A}_{\boldsymbol{x}}}(-1)^{N_{\boldsymbol{x}}-N_{\boldsymbol{y}}}\sum_{m=1}^{\infty}\kappa_m^{(t)}\sum_{\boldsymbol{h}\in\mathscr{H}}\log p_{\boldsymbol{h}}\sum_{\substack{\boldsymbol{m}:|\boldsymbol{m}|=m\\ m_{\boldsymbol{i}}=0 \text{ if } \boldsymbol{i}\notin A_{\boldsymbol{y}}}}m_{\boldsymbol{h}}\binom{m}{\boldsymbol{m}}\boldsymbol{p}_t^{\boldsymbol{m}} + \tilde{A}_t. \end{aligned} \tag{A.11c}$$

To simplify the last row above, we introduce the notation $\boldsymbol{m}_{-\boldsymbol{h}}$, which is the MOI vector $\boldsymbol{m}$ with the component of haplotype $\boldsymbol{h}$ reduced by 1, i.e., $m_{\boldsymbol{h}}$ is replaced by $m_{\boldsymbol{h}}-1$. Therefore, by rearranging the binomial coefficient $\binom{m}{\boldsymbol{m}}$ we have

$$\begin{aligned} P(\boldsymbol{x}\,|\,\boldsymbol{\theta}_t)Q_{\boldsymbol{x}}(\boldsymbol{\theta}\,|\,\boldsymbol{\theta}_t) = &- P(\boldsymbol{x}\,|\,\boldsymbol{\theta}_t)\log(e^{\lambda}-1) \\ &+ \sum_{\boldsymbol{y}\in\mathscr{A}_{\boldsymbol{x}}}(-1)^{N_{\boldsymbol{x}}-N_{\boldsymbol{y}}}\sum_{m=1}^{\infty}m\kappa_m^{(t)}\Big(\sum_{\boldsymbol{i}\in A_{\boldsymbol{y}}}p_{\boldsymbol{i}}^{(t)}\Big)^{m}\log\lambda \\ &+ \sum_{\boldsymbol{y}\in\mathscr{A}_{\boldsymbol{x}}}(-1)^{N_{\boldsymbol{x}}-N_{\boldsymbol{y}}}\sum_{\boldsymbol{h}\in\mathscr{H}}p_{\boldsymbol{h}}^{(t)}\log p_{\boldsymbol{h}}\sum_{m=1}^{\infty}\kappa_m^{(t)}\sum_{\substack{\boldsymbol{m}:|\boldsymbol{m}|=m\\ m_{\boldsymbol{i}}=0 \text{ if } \boldsymbol{i}\notin A_{\boldsymbol{y}}\\ m_{\boldsymbol{h}}>0}}\binom{m-1}{\boldsymbol{m}_{-\boldsymbol{h}}}m\boldsymbol{p}_t^{\boldsymbol{m}_{-\boldsymbol{h}}} + \tilde{A}_t. \end{aligned}$$

Rearrangement in the middle line and using the multinomial theorem (together with the fact that $\boldsymbol{h} \in A_{\boldsymbol{y}}$) in the last line yields

$$\begin{aligned} P(\boldsymbol{x}\,|\,\boldsymbol{\theta}_t)Q_{\boldsymbol{x}}(\boldsymbol{\theta}\,|\,\boldsymbol{\theta}_t) =& - P(\boldsymbol{x}\,|\,\boldsymbol{\theta}_t)\log\left(e^{\lambda}-1\right) \\ &+ \sum_{\boldsymbol{y}\in\mathscr{A}_{\boldsymbol{x}}}(-1)^{N_{\boldsymbol{x}}-N_{\boldsymbol{y}}}\sum_{\boldsymbol{h}\in A_{\boldsymbol{y}}} p_{\boldsymbol{h}}^{(t)}\sum_{m=1}^{\infty} m\kappa_m^{(t)}\Big(\sum_{\boldsymbol{i}\in A_{\boldsymbol{y}}} p_{\boldsymbol{i}}^{(t)}\Big)^{m-1}\log\lambda \\ &+ \sum_{\boldsymbol{y}\in\mathscr{A}_{\boldsymbol{x}}}(-1)^{N_{\boldsymbol{x}}-N_{\boldsymbol{y}}}\sum_{\boldsymbol{h}\in\mathscr{H}} p_{\boldsymbol{h}}^{(t)}\log p_{\boldsymbol{h}}\sum_{m=1}^{\infty} m\kappa_m^{(t)}\Big(\sum_{\boldsymbol{i}\in A_{\boldsymbol{y}}} p_{\boldsymbol{i}}^{(t)}\Big)^{m-1} + \tilde{A}_t. \end{aligned} \tag{A.11d}$$

Inside the sums in the two last lines of the above equation we recognize derivatives. The fact that $\boldsymbol{h} \in A_{\boldsymbol{y}}$ and the definition of the PGF lead to

$$\begin{aligned} \sum_{m=1}^{\infty} m\kappa_m^{(t)}\Big(\sum_{\boldsymbol{i}\in A_{\boldsymbol{y}}} p_{\boldsymbol{i}}^{(t)}\Big)^{m-1} &= \sum_{m=1}^{\infty}\kappa_m^{(t)}\frac{\partial}{\partial p_{\boldsymbol{h}}^{(t)}}\Big(\sum_{\boldsymbol{i}\in A_{\boldsymbol{y}}} p_{\boldsymbol{i}}^{(t)}\Big)^{m} \\ &= \frac{\partial}{\partial p_{\boldsymbol{h}}^{(t)}}\sum_{m=1}^{\infty}\kappa_m^{(t)}\Big(\sum_{\boldsymbol{i}\in A_{\boldsymbol{y}}} p_{\boldsymbol{i}}^{(t)}\Big)^{m} \\ &= \frac{\partial}{\partial p_{\boldsymbol{h}}^{(t)}} G\Big(\sum_{\boldsymbol{i}\in A_{\boldsymbol{y}}} p_{\boldsymbol{i}}^{(t)}\Big). \end{aligned} \tag{A.11e}$$

Combination of (A.11e) and (A.11c) gives

$$\begin{aligned} P(\boldsymbol{x}\,|\,\boldsymbol{\theta}_t)Q_{\boldsymbol{x}}(\boldsymbol{\theta}\,|\,\boldsymbol{\theta}_t) =& - P(\boldsymbol{x}\,|\,\boldsymbol{\theta}_t)\log\left(e^{\lambda}-1\right) \\ &+ \sum_{\boldsymbol{y}\in\mathscr{A}_{\boldsymbol{x}}}(-1)^{N_{\boldsymbol{x}}-N_{\boldsymbol{y}}}\sum_{\boldsymbol{h}\in A_{\boldsymbol{y}}} p_{\boldsymbol{h}}^{(t)}\frac{\partial}{\partial p_{\boldsymbol{h}}^{(t)}} G_{\lambda_t}\Big(\sum_{\boldsymbol{i}\in A_{\boldsymbol{y}}} p_{\boldsymbol{i}}^{(t)}\Big)\log\lambda \\ &+ \sum_{\boldsymbol{y}\in\mathscr{A}_{\boldsymbol{x}}}(-1)^{N_{\boldsymbol{x}}-N_{\boldsymbol{y}}}\sum_{\boldsymbol{h}\in\mathscr{H}} p_{\boldsymbol{h}}^{(t)}\log p_{\boldsymbol{h}}\frac{\partial}{\partial p_{\boldsymbol{h}}^{(t)}} G_{\lambda_t}\Big(\sum_{\boldsymbol{i}\in A_{\boldsymbol{y}}} p_{\boldsymbol{i}}^{(t)}\Big) + \tilde{A}_t, \end{aligned} \tag{A.11f}$$

where the subscript λ_t in G_{λ_t} emphasizes the dependency of the probability generating function G on λ_t in iteration t. Therefore,

$$\begin{aligned} Q_{\boldsymbol{x}}(\boldsymbol{\theta}\,|\,\boldsymbol{\theta}_t) =& -\log\left(e^{\lambda}-1\right) \\ &+ \frac{\sum_{\boldsymbol{y}\in\mathscr{A}_{\boldsymbol{x}}}(-1)^{N_{\boldsymbol{x}}-N_{\boldsymbol{y}}}\sum_{\boldsymbol{h}\in A_{\boldsymbol{y}}} p_{\boldsymbol{h}}^{(t)}\frac{\partial}{\partial p_{\boldsymbol{h}}^{(t)}} G_{\lambda_t}\Big(\sum_{\boldsymbol{i}\in A_{\boldsymbol{y}}} p_{\boldsymbol{i}}^{(t)}\Big)\log\lambda}{P(\boldsymbol{x}\,|\,\boldsymbol{\theta}_t)} \\ &+ \frac{\sum_{\boldsymbol{y}\in\mathscr{A}_{\boldsymbol{x}}}(-1)^{N_{\boldsymbol{x}}-N_{\boldsymbol{y}}}\sum_{\boldsymbol{h}\in\mathscr{H}} p_{\boldsymbol{h}}^{(t)}\log p_{\boldsymbol{h}}\frac{\partial}{\partial p_{\boldsymbol{h}}^{(t)}} G_{\lambda_t}\Big(\sum_{\boldsymbol{i}\in A_{\boldsymbol{y}}} p_{\boldsymbol{i}}^{(t)}\Big)}{P(\boldsymbol{x}\,|\,\boldsymbol{\theta}_t)} + \tilde{\tilde{A}}_t, \end{aligned} \tag{A.12a}$$

where $\tilde{\tilde{A}}_t = \dfrac{\tilde{A}_t}{P(\boldsymbol{x}\,|\,\boldsymbol{\theta}_t)}$. Note that

$$\frac{\partial}{\partial p_{\boldsymbol{h}}^{(t)}} G_{\lambda_t}\left(\sum_{\boldsymbol{i}\in A_{\boldsymbol{y}}} p_{\boldsymbol{i}}^{(t)}\right) = G'_{\lambda_t}\left(\sum_{\boldsymbol{i}\in A_{\boldsymbol{y}}} p_{\boldsymbol{i}}^{(t)}\right) I_{A_{\boldsymbol{y}}}(\boldsymbol{h}), \tag{A.12b}$$

where

$$I_{A_{\boldsymbol{y}}}(\boldsymbol{h}) = \begin{cases} 1 & \text{if } \boldsymbol{h} \in Ay, \\ 0 & \text{if } \boldsymbol{h} \notin Ay. \end{cases} \tag{A.12c}$$

Using this fact and the definition of $P(\boldsymbol{x}\,|\,\boldsymbol{\theta}_t)$ in (8c) yields,

$$\begin{aligned} Q_{\boldsymbol{x}}(\boldsymbol{\theta}\,|\,\boldsymbol{\theta}_t) = &-\log(e^{\lambda}-1) \\ &+ \frac{\sum\limits_{\boldsymbol{y}\in\mathscr{A}_{\boldsymbol{x}}} (-1)^{N_{\boldsymbol{x}}-N_{\boldsymbol{y}}} \sum\limits_{\boldsymbol{h}\in A_{\boldsymbol{y}}} p_{\boldsymbol{h}}^{(t)} G'_{\lambda_t}\left(\sum\limits_{\boldsymbol{i}\in A_{\boldsymbol{y}}} p_{\boldsymbol{i}}^{(t)}\right)\log\lambda}{\sum\limits_{\boldsymbol{y}\in\mathscr{A}_{\boldsymbol{x}}} (-1)^{N_{\boldsymbol{x}}-N_{\boldsymbol{y}}} G_{\lambda_t}\big(\sum\limits_{\boldsymbol{i}\in A_{\boldsymbol{y}}} p_{\boldsymbol{i}}^{(t)}\big)} \\ &+ \frac{\sum\limits_{\boldsymbol{y}\in\mathscr{A}_{\boldsymbol{x}}} (-1)^{N_{\boldsymbol{x}}-N_{\boldsymbol{y}}} \sum\limits_{\boldsymbol{h}\in\mathscr{H}} p_{\boldsymbol{h}}^{(t)} \log p_{\boldsymbol{h}} G'_{\lambda_t}\left(\sum\limits_{\boldsymbol{i}\in A_{\boldsymbol{y}}} p_{\boldsymbol{i}}^{(t)}\right) I_{A_{\boldsymbol{y}}}(\boldsymbol{h})}{\sum\limits_{\boldsymbol{y}\in\mathscr{A}_{\boldsymbol{x}}} (-1)^{N_{\boldsymbol{x}}-N_{\boldsymbol{y}}} G_{\lambda_t}\left(\sum\limits_{\boldsymbol{i}\in A_{\boldsymbol{y}}} p_{\boldsymbol{i}}^{(t)}\right)} + \tilde{\tilde{A}}_t. \end{aligned} \tag{A.12d}$$

The Q-function in (A.9c) becomes

$$\begin{aligned} Q(\boldsymbol{\theta}\,|\,\boldsymbol{\theta}_t) = &-N\log\left(e^{\lambda}-1\right) \\ &+ \sum_{\boldsymbol{x}\in\mathscr{O}} n_{\boldsymbol{x}} \frac{\sum\limits_{\boldsymbol{y}\in\mathscr{A}_{\boldsymbol{x}}} (-1)^{N_{\boldsymbol{x}}-N_{\boldsymbol{y}}} \sum\limits_{\boldsymbol{h}\in A_{\boldsymbol{y}}} p_{\boldsymbol{h}}^{(t)} G'_{\lambda_t}\left(\sum\limits_{\boldsymbol{i}\in A_{\boldsymbol{y}}} p_{\boldsymbol{i}}^{(t)}\right)\log\lambda}{\sum\limits_{\boldsymbol{y}\in\mathscr{A}_{\boldsymbol{x}}} (-1)^{N_{\boldsymbol{x}}-N_{\boldsymbol{y}}} G_{\lambda_t}\left(\sum\limits_{\boldsymbol{i}\in A_{\boldsymbol{y}}} p_{\boldsymbol{i}}^{(t)}\right)} \\ &+ \sum_{\boldsymbol{x}\in\mathscr{O}} n_{\boldsymbol{x}} \frac{\sum\limits_{\boldsymbol{y}\in\mathscr{A}_{\boldsymbol{x}}} (-1)^{N_{\boldsymbol{x}}-N_{\boldsymbol{y}}} \sum\limits_{\boldsymbol{h}\in\mathscr{H}} p_{\boldsymbol{h}}^{(t)} \log p_{\boldsymbol{h}} G'_{\lambda_t}\left(\sum\limits_{\boldsymbol{i}\in A_{\boldsymbol{y}}} p_{\boldsymbol{i}}^{(t)}\right) I_{A_{\boldsymbol{y}}}(\boldsymbol{h})}{\sum\limits_{\boldsymbol{y}\in\mathscr{A}_{\boldsymbol{x}}} (-1)^{N_{\boldsymbol{x}}-N_{\boldsymbol{y}}} G_{\lambda_t}\left(\sum\limits_{\boldsymbol{i}\in A_{\boldsymbol{y}}} p_{\boldsymbol{i}}^{(t)}\right)} + \tilde{\tilde{A}}_t. \end{aligned} \tag{A.12e}$$

Reordering the sums in the third term yields

$$
\begin{aligned}
Q(\boldsymbol{\theta}\,|\,\boldsymbol{\theta}_t) = &-N\log\left(e^{\lambda}-1\right)\\
&+\sum_{\boldsymbol{x}\in\mathscr{O}} n_{\boldsymbol{x}} \frac{\sum\limits_{\boldsymbol{y}\in\mathscr{A}_{\boldsymbol{x}}}(-1)^{N_{\boldsymbol{x}}-N_{\boldsymbol{y}}} \sum\limits_{\boldsymbol{h}\in A_{\boldsymbol{y}}} p_{\boldsymbol{h}}^{(t)} G'_{\lambda_t}\left(\sum\limits_{\boldsymbol{i}\in A_{\boldsymbol{y}}} p_{\boldsymbol{i}}^{(t)}\right)\log\lambda}{\sum\limits_{\boldsymbol{y}\in\mathscr{A}_{\boldsymbol{x}}}(-1)^{N_{\boldsymbol{x}}-N_{\boldsymbol{y}}} G_{\lambda_t}\left(\sum\limits_{\boldsymbol{i}\in A_{\boldsymbol{y}}} p_{\boldsymbol{i}}^{(t)}\right)} \\
&+\sum_{\boldsymbol{h}\in\mathscr{H}} \frac{\sum\limits_{\boldsymbol{x}\in\mathscr{O}} n_{\boldsymbol{x}} \sum\limits_{\boldsymbol{y}\in\mathscr{A}_{\boldsymbol{x}}}(-1)^{N_{\boldsymbol{x}}-N_{\boldsymbol{y}}} p_{\boldsymbol{h}}^{(t)} \log p_{\boldsymbol{h}} G'_{\lambda_t}\left(\sum\limits_{\boldsymbol{i}\in A_{\boldsymbol{y}}} p_{\boldsymbol{i}}^{(t)}\right) I_{A_{\boldsymbol{y}}}(\boldsymbol{h})}{\sum\limits_{\boldsymbol{y}\in\mathscr{A}_{\boldsymbol{x}}}(-1)^{N_{\boldsymbol{x}}-N_{\boldsymbol{y}}} G_{\lambda_t}\left(\sum\limits_{\boldsymbol{i}\in A_{\boldsymbol{y}}} p_{\boldsymbol{i}}^{(t)}\right)} + \tilde{\tilde{A}}_t.
\end{aligned}
\tag{A.12f}
$$

We let

$$
B_t = \sum_{\boldsymbol{x}\in\mathscr{O}} n_{\boldsymbol{x}} \frac{\sum\limits_{\boldsymbol{y}\in\mathscr{A}_{\boldsymbol{x}}}(-1)^{N_{\boldsymbol{x}}-N_{\boldsymbol{y}}} \sum\limits_{\boldsymbol{h}\in A_{\boldsymbol{y}}} p_{\boldsymbol{h}}^{(t)} G'_{\lambda_t}\left(\sum\limits_{\boldsymbol{i}\in A_{\boldsymbol{y}}} p_{\boldsymbol{i}}^{(t)}\right)}{\sum\limits_{\boldsymbol{y}\in\mathscr{A}_{\boldsymbol{x}}}(-1)^{N_{\boldsymbol{x}}-N_{\boldsymbol{y}}} G_{\lambda_t}\left(\sum\limits_{\boldsymbol{i}\in A_{\boldsymbol{y}}} p_{\boldsymbol{i}}^{(t)}\right)}, \tag{A.12g}
$$

and

$$
C_{\boldsymbol{h}}^{(t)} = p_{\boldsymbol{h}}^{(t)} \sum_{\boldsymbol{x}\in\mathscr{O}} n_{\boldsymbol{x}} \frac{\sum\limits_{\boldsymbol{y}\in\mathscr{A}_{\boldsymbol{x}}}(-1)^{N_{\boldsymbol{x}}-N_{\boldsymbol{y}}} G'_{\lambda_t}\left(\sum\limits_{\boldsymbol{i}\in A_{\boldsymbol{y}}} p_{\boldsymbol{i}}^{(t)}\right) I_{A_{\boldsymbol{y}}}(\boldsymbol{h})}{\sum\limits_{\boldsymbol{y}\in\mathscr{A}_{\boldsymbol{x}}}(-1)^{N_{\boldsymbol{x}}-N_{\boldsymbol{y}}} G_{\lambda_t}\left(\sum\limits_{\boldsymbol{i}\in A_{\boldsymbol{y}}} p_{\boldsymbol{i}}^{(t)}\right)}, \tag{A.12h}
$$

such that the Q-function is rewritten as

$$
Q(\boldsymbol{\theta}\,|\,\boldsymbol{\theta}_t) = -N\log\left(e^{\lambda}-1\right) + B_t\log\lambda + \sum_{\boldsymbol{h}\in\mathscr{H}} C_{\boldsymbol{h}}^{(t)} \log p_{\boldsymbol{h}} + \tilde{\tilde{A}}_t. \tag{A.12i}
$$

Maximization (M)-step

The maximization of the Q-function is performed during the M-step of the EM-algorithm. The estimates $\hat{\boldsymbol{\theta}}$ of the model parameters at iteration $t+1$ are obtained as

$$
\hat{\boldsymbol{\theta}} = \arg\max_{\boldsymbol{\theta}} Q(\boldsymbol{\theta}\,|\,\boldsymbol{\theta}_t).
$$

We maximize the Q-function using the method of Lagrange-multipliers under the constraint $\sum_{\boldsymbol{h}\in\mathscr{H}} p_{\boldsymbol{h}} = 1$. The Lagrangian function is denoted by L such that

$$L(\boldsymbol{\theta}\,|\,\boldsymbol{\theta}_t) = Q(\boldsymbol{\theta}\,|\,\boldsymbol{\theta}_t) - \gamma\Big(\sum_{\boldsymbol{h}\in\mathscr{H}} p_{\boldsymbol{h}} - 1\Big),$$

where γ is the Lagrange multiplier. The parameters estimates $\hat{\boldsymbol{\theta}}$ at iteration $t+1$ of the EM-algorithm are such that

$$\nabla L(\hat{\boldsymbol{\theta}}\,|\,\boldsymbol{\theta}_t) = \mathbf{0}.$$

The Lagrangian function is given by

$$L(\boldsymbol{\theta}\,|\,\boldsymbol{\theta}_t) = -N\log\left(e^{\lambda}-1\right) + B_t\log\lambda + \sum_{\boldsymbol{h}\in\mathscr{H}} C_{\boldsymbol{h}}^{(t)}\log p_{\boldsymbol{h}} - \gamma\Big(\sum_{\boldsymbol{h}\in\mathscr{H}} p_{\boldsymbol{h}} - 1\Big) + \tilde{\tilde{A}}_t, \tag{A.13}$$

and the components of its gradient are

$$\frac{\partial L(\boldsymbol{\theta}\,|\,\boldsymbol{\theta}_t)}{\partial\lambda} = -\frac{Ne^{\lambda}}{e^{\lambda}-1} + \frac{B_t}{\lambda}, \tag{A.14a}$$

$$\frac{\partial L(\boldsymbol{\theta}\,|\,\boldsymbol{\theta}_t)}{\partial p_{\boldsymbol{h}}} = C_{\boldsymbol{h}}^{(t)}\frac{1}{p_{\boldsymbol{h}}} - \gamma, \tag{A.14b}$$

$$\frac{\partial L(\boldsymbol{\theta}\,|\,\boldsymbol{\theta}_t)}{\partial\gamma} = -\sum_{\boldsymbol{h}\in\mathscr{H}} p_{\boldsymbol{h}} + 1. \tag{A.14c}$$

To obtain the estimates $\hat{\boldsymbol{\theta}}$, we equate the above components of the gradient to zero. Equating (A.14b) to 0 gives

$$p_{\boldsymbol{h}} = \frac{C_{\boldsymbol{h}}^{(t)}}{\gamma}.$$

Substituting this expression into (A.14c) and equating the resulting term to 0 yields

$$\gamma = \sum_{\boldsymbol{h}\in\mathscr{H}} C_{\boldsymbol{h}}^{(t)}.$$

Combining the last two equations gives the frequency of haplotype $\boldsymbol{h}$ at iteration $t+1$, namely

$$p_{\boldsymbol{h}}^{(t+1)} = \frac{C_{\boldsymbol{h}}^{(t)}}{\sum_{\boldsymbol{h}\in\mathscr{H}} C_{\boldsymbol{h}}^{(t)}}. \tag{A.15}$$

The Poisson parameter λ at step $t+1$ is obtained by equating (A.14a) to 0, i.e.,

$$-\frac{Ne^{\lambda}}{e^{\lambda}-1}+\frac{B_t}{\lambda}=0.$$

This non-linear equation has no closed solution and needs to be solved numerically. We do so by the 1-dimensional Newton-Raphson method. Let the function f be defined as

$$f(\lambda):=\frac{\lambda e^{\lambda}}{e^{\lambda}-1}-\frac{B_t}{N}, \tag{A.16a}$$

and the 1-dimensional Newton-Raphson method yields the recursion equation

$$\lambda_{\tau+1}=\lambda_{\tau}-\frac{f(\lambda_{\tau})}{f'(\lambda_{\tau})}, \tag{A.16b}$$

where

$$f'(\lambda)=\frac{(1+\lambda)(1-e^{-\lambda})-\lambda}{(1-e^{-\lambda})^2}. \tag{A.16c}$$

Therefore, the recursion equation is given by

$$\lambda_{\tau+1}=\lambda_{\tau}-\frac{\lambda_{\tau}-\frac{B_t}{N}(1-e^{-\lambda_{\tau}})}{1+\lambda_{\tau}-\frac{\lambda_{\tau}}{1-e^{-\lambda_{\tau}}}}. \tag{A.16d}$$

The value of the Poisson parameter λ_{t+1} at iteration $t+1$ of the EM-algorithm is the limit of the recursion (A.16d).

1.3 The EM-algorithm using a plug-in estimate for the Poisson parameter

If one prefers to use a plug-in estimate for the Poisson parameter λ, it is straightforward to adapt the EM-algorithm. In fact, most of the derivations in 1.2 remain valid and need just little adjustments. In particular, $\lambda_t=\lambda=\lambda_{\text{plug-in}}$.

In particular, the Q-function becomes

$$Q(\boldsymbol{\theta}\,|\,\boldsymbol{\theta}_t)=\sum_{\boldsymbol{h}\in\mathscr{H}}C_{\boldsymbol{h}}^{(t)}\log p_{\boldsymbol{h}}+A_t^{*}, \tag{A.17}$$

where $\boldsymbol{\theta} = \boldsymbol{p}$, $\boldsymbol{\theta}_t = \boldsymbol{p}_t$,

$$C_{\boldsymbol{h}}^{(t)} = p_{\boldsymbol{h}}^{(t)} \sum_{\boldsymbol{x} \in \mathscr{O}} n_{\boldsymbol{x}} \frac{\sum\limits_{\boldsymbol{y} \in \mathscr{A}_{\boldsymbol{x}}} (-1)^{N_{\boldsymbol{x}} - N_{\boldsymbol{y}}} G'_{\lambda_{\text{plug-in}}} \left(\sum\limits_{\boldsymbol{i} \in A_{\boldsymbol{y}}} p_{\boldsymbol{i}}^{(t)} \right) I_{A_{\boldsymbol{y}}}(\boldsymbol{h})}{\sum\limits_{\boldsymbol{y} \in \mathscr{A}_{\boldsymbol{x}}} (-1)^{N_{\boldsymbol{x}} - N_{\boldsymbol{y}}} G_{\lambda_{\text{plug-in}}} \left(\sum\limits_{\boldsymbol{i} \in A_{\boldsymbol{y}}} p_{\boldsymbol{i}}^{(t)} \right)}, \tag{A.18}$$

and A_t^* is independent of the parameters $\boldsymbol{\theta}$.

The maximization step is adapted similarly and leads to the iteration

$$p_{\boldsymbol{h}}^{(t+1)} = \frac{C_{\boldsymbol{h}}^{(t)}}{\sum\limits_{\boldsymbol{h} \in \mathscr{H}} C_{\boldsymbol{h}}^{(t)}}. \tag{A.19}$$

1.4 Samplewise MOI

The probability of MOI $= m$ given infection $\boldsymbol{x}$ is

$$P(\text{MOI} = m|\boldsymbol{x}) = \frac{P(\boldsymbol{x}, m)}{P(\boldsymbol{x})}, \tag{A.20}$$

where $P(\boldsymbol{x}, m)$ is defined in (6b) and $P(\boldsymbol{x})$ in (6c). Hence, after deriving the MLEs $\hat{\theta}$, the probability $P(\text{MOI} = m|\boldsymbol{x})$ becomes

$$P(\text{MOI} = m|\boldsymbol{x}; \hat{\theta}) = P(\text{MOI} = m|\boldsymbol{x}; \hat{\lambda}, \hat{\boldsymbol{p}}) = \frac{\hat{\kappa}_m \sum\limits_{\boldsymbol{m} \in M_{\boldsymbol{x}}^{(m)}} \binom{m}{\boldsymbol{m}} \hat{\boldsymbol{p}}^{\boldsymbol{m}}}{\sum\limits_{m=1}^{\infty} \hat{\kappa}_m \sum\limits_{\boldsymbol{m} \in M_{\boldsymbol{x}}^{(m)}} \binom{m}{\boldsymbol{m}} \hat{\boldsymbol{p}}^{\boldsymbol{m}}}, \tag{A.21}$$

and, similarly as above, the inclusion-exclusion principle and the definition of the generating function yield

$$P(\text{MOI} = m|\boldsymbol{x}; \hat{\lambda}, \hat{\boldsymbol{p}}) = \frac{\hat{\kappa}_m \sum\limits_{\boldsymbol{y} \in \mathscr{A}_{\boldsymbol{x}}} (-1)^{N_{\boldsymbol{x}} - N_{\boldsymbol{y}}} \left(\sum\limits_{\boldsymbol{h} \in A_{\boldsymbol{y}}} \hat{p}_{\boldsymbol{h}} \right)^m}{\sum\limits_{\boldsymbol{y} \in \mathscr{A}_{\boldsymbol{x}}} (-1)^{N_{\boldsymbol{x}} - N_{\boldsymbol{y}}} G_{\hat{\lambda}} \left(\sum\limits_{\boldsymbol{h} \in A_{\boldsymbol{y}}} \hat{p}_{\boldsymbol{h}} \right)}. \tag{A.22}$$
